# PGAM1, regulated by miR-3614-5p, functions as an oncogene by activating transforming growth factor-β (TGF-β) signaling in the progression of non-small cell lung carcinoma

**DOI:** 10.1038/s41419-020-02900-4

**Published:** 2020-08-27

**Authors:** Fangfang Li, Hao Yang, Tiandong Kong, Shanshan Chen, Ping Li, Lu Chen, Jiuling Cheng, Guangying Cui, Guojun Zhang

**Affiliations:** 1 Department of Respiratory Medicine, The First Affiliated Hospital of Zhengzhou University, Zhengzhou, China; 2 Department of Orthopedics, The First Affiliated Hospital of Zhengzhou University, Zhengzhou, China; 3 Department of Oncology, The Third People’s Hospital of Zhengzhou, Zhengzhou, 450000 China; 4 Precision Medicine Center, The First Affiliated Hospital of Zhengzhou University, Zhengzhou, China

**Keywords:** Non-small-cell lung cancer, Cell invasion

## Abstract

Phosphoglycerate mutase 1 (PGAM1) is a recently identified key catalytic enzyme in aerobic glycolysis. Recent literature has documented that dysregulated PGAM1 expression is associated with tumorigenesis in various cancers. However, the expression status and biological function of PGAM1 in non-small-cell lung cancer (NSCLC) are poorly elucidated. In this study, we found that PGAM1 was overexpressed in NSCLC tissues and that high expression of PGAM1 was associated with poor prognosis in NSCLC patients. Functionally, gain- and loss-of-function analysis showed that PGAM1 promoted proliferation and invasion in vitro, and facilitated tumor growth in vivo. Mechanistically, the transforming growth factor-β (TGF-β) signaling pathway was also markedly impaired in response to PGAM1 silencing. Additionally, we verified that PGAM1 was inhibited by miR-3614-5p via direct targeting of its 3’-untranslated regions in a hypoxia-independent manner. Furthermore, overexpression of miR-3614-5p attenuated NSCLC cell proliferation and invasion, and these effects could be partially reversed by reintroduction of PGAM1. Conclusively, our results suggest that the miR-3614-5p/PGAM1 axis plays a critical role during the progression of NSCLC, and these findings may provide a potential target for the development of therapeutic strategies for NSCLC patients.

## Background

Globally, lung cancer is the most prevalent malignant tumor and accounts for approximately 27% of all cancer-related deaths^1^. Lung cancer is generally divided into two histological subtypes: small cell lung cancer and non-small-cell lung cancer (NSCLC), which accounts for approximately 80% of all lung cancers^2^. Despite great effort has been made, NSCLC patients with advanced-stage disease exhibit high mortality after resection due to metastatic lesions or local recurrence^3^. Thus, elucidating the molecular mechanisms underlying NSCLC progression and metastasis is crucial for the treatment of the disease.

Recently, phosphoglycerate mutase 1 (PGAM1), a key aerobic glycolysis enzyme, has been identified to be frequently upregulated and involved in the tumorigenesis of different types of human cancers, such as hepatocellular carcinoma, oral squamous cell carcinoma, glioma, urothelial bladder cancer, renal clear cell carcinoma and colorectal cancer^4–9^. In addition, proteomic analysis results indicated that PGAM1 was highly expressed in NSCLC^10^. Moreover, Sun et al. revealed that high PGAM1 expression was associated with reduced patient survival, and PGAM1 silencing suppressed aggressive tumor phenotypes and mTOR-dependent glycolysis of NSCLC cell^11^. Interestingly another novel PGAM1 allosteric inhibitor, HKB99, has been reported to suppress tumor growth and metastasis and overcome drug resistance in NSCLC^12^. Together, these work provides a preclinical proof of concept for PGAM1 as a novel candidate target in NSCLC. However, the relationship between PGAM1 expression and prognosis in NSCLC and the functional role and regulatory network of PGAM1 remain largely unknown.

MicroRNAs (miRs, miRNAs) are small noncoding single-stranded RNAs that lack protein-encoding functions. MiRNAs can bind to the 3′ untranslated regions (3′-UTRs) of their target mRNAs to suppress protein expression^13^. Mounting evidence has indicated that miRNAs play crucial roles in the progression of most human cancers by acting as oncogenic RNAs or tumor suppressors^14^. MiR-3614-5p, with a length of 24 nt, is located on chromosome 17q22. It has been reported that miR-3614-5p can combat dengue virus by regulating the action of adenosine deaminase acting on RNA 1 (ADAR1) in human macrophages^15^. Another report indicated a close relationship between dysregulated miR-3614-5p expression and autoimmune disease risk^16^. Additionally, overexpression of miR-3614-5p dramatically inhibited the proliferation capacity of breast cancer cells^17^. Bioinformatics analysis indicated that miR-3614-5p may suppress WNT signal pathway through targeting NFATC2 in NSCLC cell^18^. Nevertheless, whether miR-3614-5p plays a role in the development and progression of NSCLC remains largely unclear.

In the present study, we demonstrated that PGAM1 was significantly upregulated in tumor tissues and associated with poor clinical prognosis of NSCLC. Functional experiments revealed that PGAM1 could promote the proliferation and invasion of NSCLC cell lines. Moreover, we discovered that PGAM1 was inhibited by miR-3614-5p and could reverse miR-3614-5p’s inhibitory effects on NSCLC tumorigenesis. An investigation of the mechanism found that the transforming growth factor-β (TGF-β) signaling pathway was regulated by the miR-3614-5p/PGAM1 axis. Finally, in vivo experiments further validated that miR-3614-5p inhibited NSCLC tumor growth by targeting PGAM1. Taken together, our findings reveal a critical role of the miR-3614-5p/PGAM1 axis in mediating TGF-β signaling pathway activation in NSCLC progression.

## Results

### PGAM1 mRNA is significantly upregulated and correlated with the prognosis of NSCLC patients

**Fig. 1:**
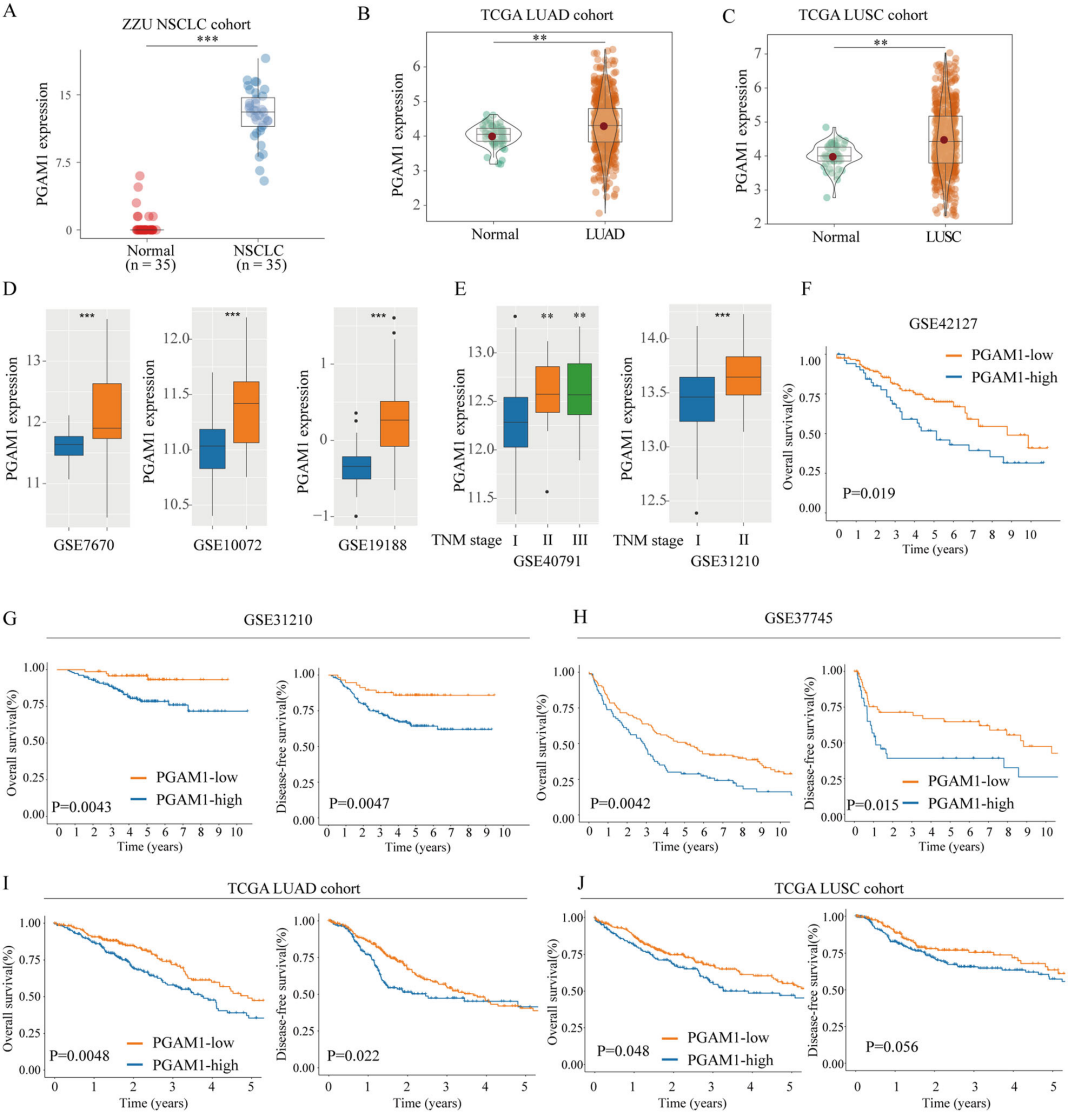
PGAM1 mRNA is upregulated in NSCLC based on TCGA analysis. PGAM1 mRNA expression levels in NSCLC tissues or non-tumor control tissues were analyzed by QT-PCR in 30 paired NSCLC specimens (**a**). PGAM1 mRNA expression levels were analyzed in LUAD and LUSC tissues from TCGA databases (**b, c**), and three independent GEO datasets (**d**). Expression of PGAM1 in different TNM stage (**e**). Overall survival (OS) and disease-free survival (DFS) rates analysis of the NSCLC patients in GSE42127 (**f**), GSE31210 (**g**), GSE37745 (**h**) and TCGA cohort (**i**, **j**). *p < 0.05, **p < 0.01, ***p < 0.001.

First, we determined that PGAM1 mRNA expression were markedly high in most solid cancer tissues through TCGA data analysis (Supplementary Fig. S1A). We also confirmed that the other isoform PGAM2 was negatively expressed in NSCLC tissues (Supplementary Fig. S1B–E). The PGAM1 mRNA levels in the NSCLC tissues lines were significantly higher than those in normal tissues, as confirmed by RT-PCR analyses in 30 paired NSCLC specimens (Fig. 1a). In addition, a similar result was also observed in the TCGA (Fig. 1b, c) and several GEO NSCLC cohorts (Fig. 1d). In addition, we found that there was a significant positive correlation between the PGAM1 and advanced TNM stage (Fig. 1d) and tumor proliferation marker (Ki-67 and PCNA) expression levels (Supplementary Fig. S2A, B). To explore the prognostic value of PGAM1, the overall survival (OS) and disease-free survival (DFS) rates were analyzed in TCGA cohorts and three additional independent GEO cohorts. The Kaplan–Meier analysis results showed that both OS and DFS time were significantly shorter in patients with high-PGAM1-expression than in patients with low-PGAM1-expression in almost all NSCLC cohorts (Fig. 1e–i). Additionally, a ROC curve was used to evaluate the diagnostic value of PGAM1, and we found that the proportion of PGAM1 under the ROC curve (AUC) was 0.79 (TCGA), 0.879 (GSE19188), 0.746 (GSE7670) and 0.774 (GSE10072), respectively (Supplementary Fig. S3A–D). Collectively, these results revealed that PGAM1 was significantly upregulated, and potentially represents a novel prognostic and diagnostic biomarker for NSCLC patients.

### Upregulation of PGAM1 protein levels predicts unfavorable prognosis in NSCLC

**Fig. 2:**
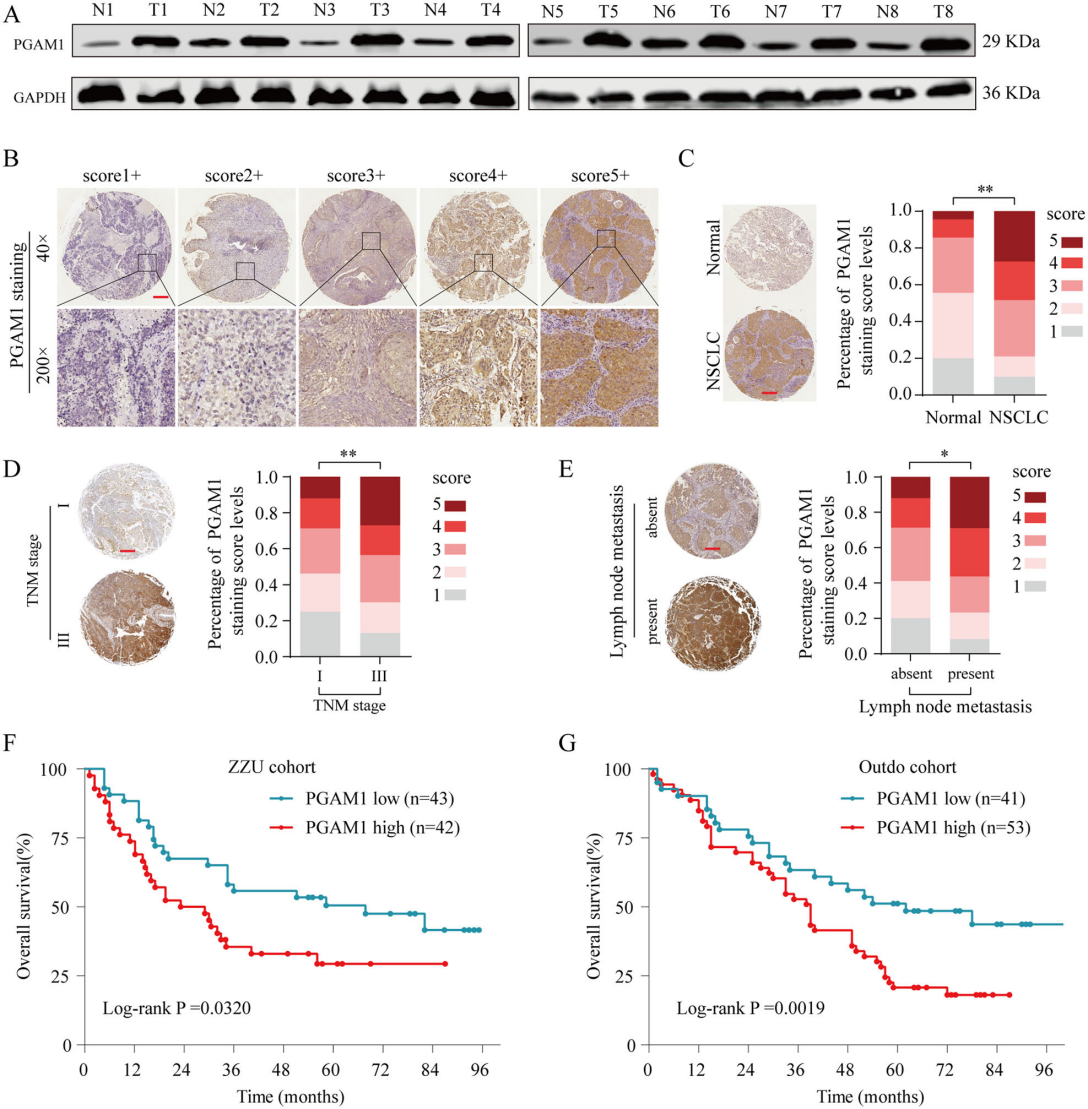
PGAM1 is upregulated in NSCLC tissues and associated with poor prognosis in TMA cohort. **a** Protein levels of PGAM1 in 8 paired NSCLC and matched adjacent non-tumor tissues were determined by Western blot assay. (N, matched adjacent non-tumor tissues; T, tumor tissues). **b** Representative expression pattern of PGAM1 with different IHC staining score. **c** Distribution of PGAM1 IHC staining score in NSCLC tissue samples or non-tumor tissues. **d, e** Representative expression pattern of PGAM1 in NSCLC tissue samples from patients with different TNM stages or with absent/present lymph node metastasis. Kaplan-Meier analysis of the OS rate in NSCLC patients in ZZU cohort (**f**) and Outdo cohort (**g**). *p < 0.05, **p < 0.01.

We further confirmed the dysregulated PGAM1 expression in NSCLC at the protein level. As shown in Fig. 2a, PGAM1 expression was dramatically increased in NSCLC tissues when compared to their normal counterparts. In addition, the relationship between PGAM1 expression and clinical prognosis in two independent NSCLC tissue microarray (TMA) cohorts (ZZU cohort and Outdo cohort) was evaluated through IHC assay. PGAM1 expression was scored from 1+ to 5+ according to the staining intensity of each specimen (Fig. 2b). The results confirmed that PGAM1 displayed markedly higher expression in NSCLC tissues than in in noncancerous tissues, and the immunoreactivity of PGAM1 was mostly distributed in both the cytoplasm and cell membrane (Fig. 2c). Moreover, we found that high PGAM1 expression was significantly correlated with advanced TNM stage and lymph node metastasis (Fig. 2d, e). Furthermore, consistent with the results observed in the public genome cohort, strong PGAM1 IHC staining was significantly associated with poor survival in both the ZZU and Outdo cohorts (Fig. 2f, g).

Furthermore, univariate and multivariate analysis suggested that PGAM1 expression, lymph node metastasis and TNM stage were independent predictors of OS in patients with NSCLC (Tables 1 and 2). Taken together, these findings strongly suggested that high PGAM1 expression status was positively associated with poor prognosis and might serve as a prognostic biomarker in NSCLC patients.

**Table 1:** Clinicopathological characteristics and expression of PGAM1 in ZZU NSCLC cohort.

	Clinicopathological features	No. of cases	PGAM1 expression		*p*-Value^a^
			Low (*n* = 43)	High (*n* = 42)	
Age(years)	≤50	36	16	20	0.331
	>50	49	27	22	
Gender	Male	55	27	28	0.708
	Female	30	16	14	
Smoking	No	32	15	17	0.594
	Yes	53	28	25	
Subtype	LUAD	48	22	26	0.317
	LUSC	37	21	16	
Lymph metastasis	No	26	18	8	**0.022**
	Yes	59	25	34	
TNM stage	I & II	51	31	20	**0.021**
	III & IV	34	12	22	
Tumor size	<5 cm	50	32	18	**0.003**
	≥5 cm	35	11	24	

*LUAD* lung adenocarcinoma, *LUSC* Lung squamous cell carcinoma. Bold values indicate statistical significance, *p* < 0.05.

**Table 2:** Correlation of clinic-pathological features with PGAM1 expression in ZZU NSCLC cohort.

	Univariate analysis			Multivariate analysis		
	HR	95% CI	*p-*Value	HR	95% CI	*p-*Value
**Univariate and multivariate analysis of overall survival in ZZU LUSC cohort (*n*** = **85)**						
Age	1.245	0.723–1.765	0.681			
Gender	0.829	0.567–1.392	0.371			
Smoking	1.023	0.1498–1.348	0.651			
Subtype	0.885	0.665–1.126	0.687			
Lymph metastasis	1.955	0.911–2.494	**0.034**	1.454	0.824–1.931	**0.047**
TNM stage	2.497	1.609–3.345	**0.012**	2.031	1.469–2.698	**0.002**
Tumor size	1.735	1.113–2.485	**0.036**	1.647	1.248–2.229	0.059
PGAM1 expression	2.043	1.984–3.412	**0.028**	1.954	1.478–2.314	**0.013**

Bold values indicate statistical significance, *p* < 0.05.

### PGAM1 promotes NSCLC cell growth, migration and invasion in vitro

**Fig. 3:**
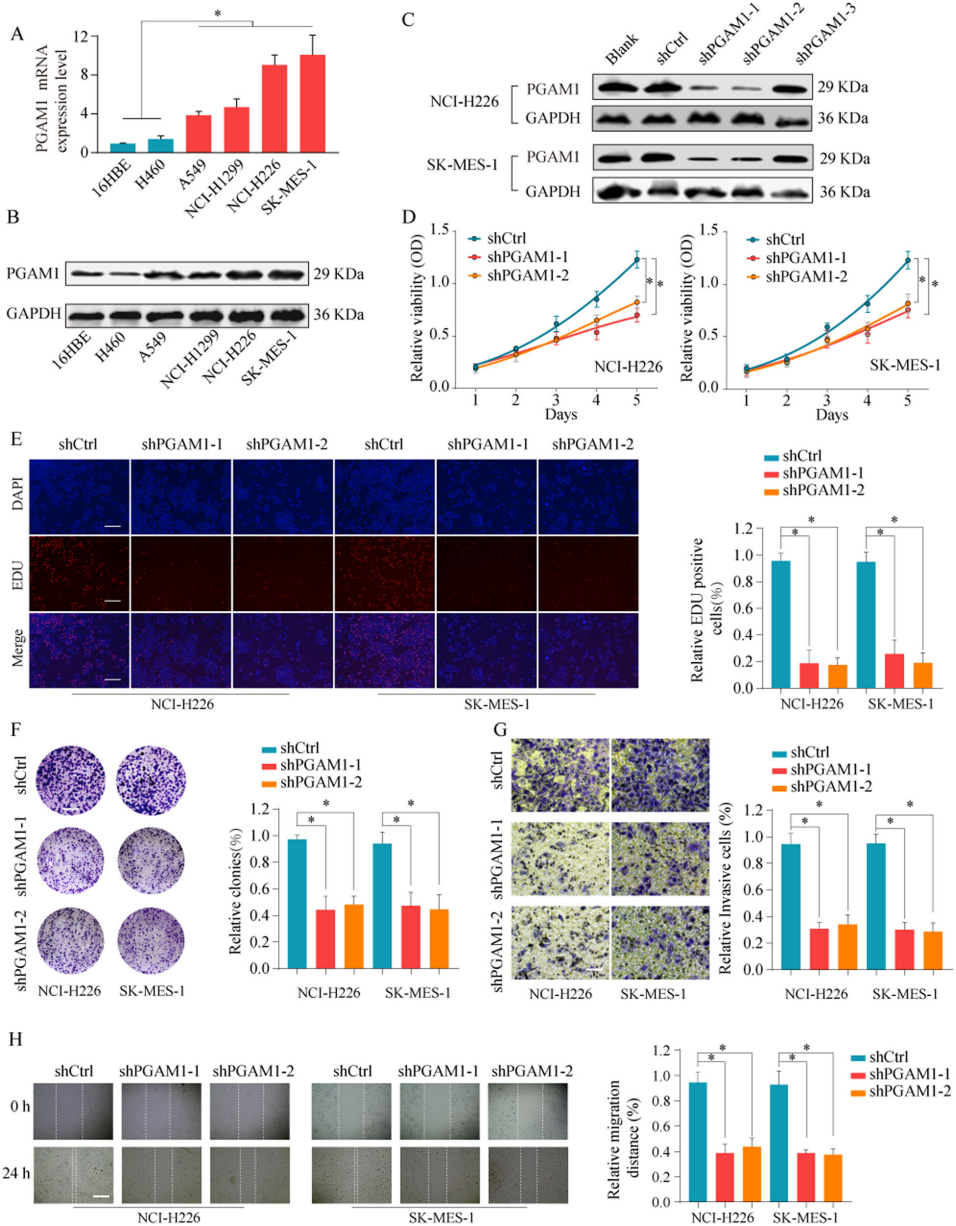
PGAM1 knockdown inhibit NSCLC cell proliferation and invasion. **a**, **b** The mRNA and protein expression levels of PGAM1 in bronchial epithelial cell 16HBE and NSCLC cell lines (A549, H1299, NCI-H226 and SK-MES-1) were analyzed by RT-qPCR (up panel) and western blot (lower panel). **c** NCI-H226 and SK-MES-1 cells were untreated (Blank), transfected with negative control (shCtrl), or transfected with shRNAs targeting PGAM1 (sh-PGAM1-1/2/3). The expression of PGAM1 protein was analyzed by western blot 48 h later. The representative result of three independent experiments was shown. Cell proliferation capability of NCI-H226 and SK-MES-1 cells transfected with shCtrl or shPGAM1-1/2 was determined by CCK-8 assay (**d**), EdU assay (**e**) and colony formation assay (**f**). Transwell experiment (**g**) and wounding healing assay (**h**) was performed to analyze the cell invasion and migration capability of NCI-H226 and SK-MES-1 cells transfected with shCtrl or shPGAM1-1/2. The results are presented as the mean ± SD. *p < 0.05, **p < 0.01 by Mann–Whitney *U*-test.

The above findings prompted us to elucidate the potential biological function of PGAM1 in NSCLC cells. Initially, we detected PGAM1 expression in NSCLC cell lines. The results showed that PGAM1 was highly expressed in NSCLC cell lines compared with normal lung cells at both the mRNA and protein levels (Fig. 3a, b). To evaluate the effects of PGAM1 on aggressive tumor phenotypes in NSCLC cells, loss- and gain-of-function experiments were conducted. First, the transfection efficacy of PGAM1 siRNA was confirmed (Fig. 3c). CCK-8 and EdU assays indicated that PGAM1 silencing significantly inhibited the proliferation capacity and DNA synthesis rate of NCI-H226 and SK-MES-1 cells (Fig. 3d, e). Similarly, colony-formation ability was reduced after PGAM1 silencing (Fig. 3f). In addition, transwell and wound healing assays indicated that the invasion and migration capacities of NSCLC cells in the PGAM1 silencing groups were markedly reduced compared to those in the control group (Fig. 3g, h).

Furthermore, to further confirm whether PGAM1 silencing stimulated cell apoptosis, we performed a TUNEL assay, and the findings suggested that knockdown of PGAM1 potently augmented the apoptosis of NCI-H226 and SK-MES-1 cells (Supplementary Fig. S4A). Consistently, PGAM1 knockdown largely reduced the expression of invasion-related proteins MMP2/7/9 (Supplementary Fig. S4B), increased the expression of the proapoptotic protein BAX, cleaved caspase 3 and cytochrome C and suppressed the expression of the anti-apoptotic protein Bcl-2 (Supplementary Fig. S4C). Conversely, PGAM1 overexpression promoted the proliferation and invasion of NSCLC cells (Supplementary Fig. S5). Collectively, these results indicated the crucial role of upregulated PGAM1 expression in facilitating NSCLC cell growth and metastasis.

### Unregulated PGAM1 enhances tumorigenicity in vivo

**Fig. 4:**
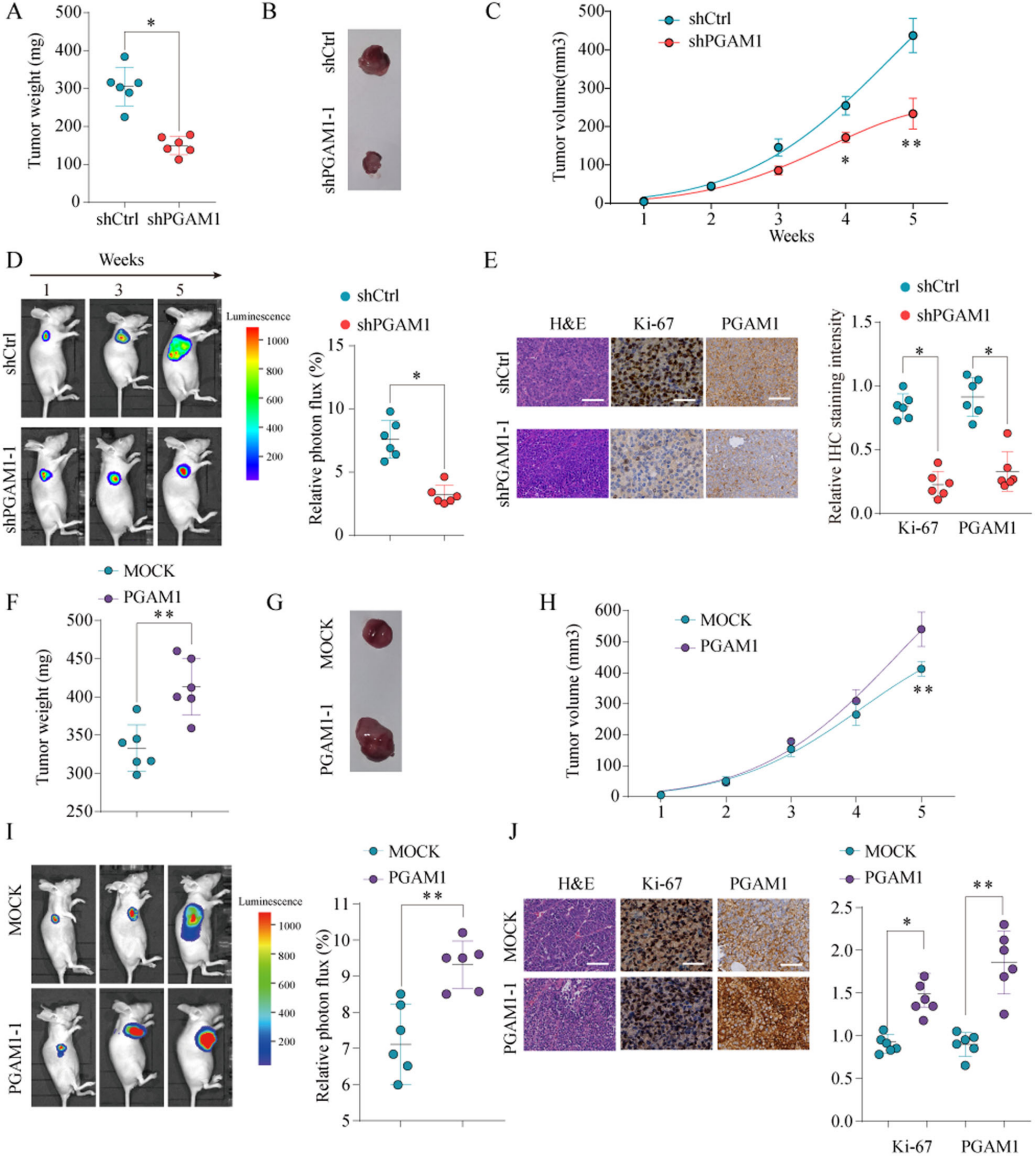
PGAM1 promotes the proliferation of NSCLC cells in vivo. A549 cells infected by lentiviral to achieve PGAM1 stable knockdown (sh-PGAM1) or PGAM1 overexpression (PGAM1) or infected by negative control (shCtrl or MOCK, respectively) were implanted into the nude mice and tumor growth was recorded. Tumor weight in nude mice was assessed at week 5 (**a**, **b**) and tumor volume (**c**) was determined based on tumor size measured every week in nude mice from shCtrl or shPGAM1 group. Representative bioluminescent images and quantification of bioluminescent imaging signal intensities in nude mice from shCtrl or shPGAM1 group (**d**). Representative images of HE staining, Ki-67 and PGAM1 IHC staining of tumor tissues obtained from nude mice from shCtrl or shPGAM1 group. Scale bar = 100 μm (**e**). Tumor weight in nude mice was assessed at week 5 (**f**, **g**) and tumor volume (**h**) was determined based on tumor size measured every week in nude mice from MOCK or PGAM1 group. Representative bioluminescent images and quantification of bioluminescent imaging signal intensities in nude mice from MOCK or PGAM1 group (**i**). Representative images of HE staining, Ki-67 and PGAM1 IHC staining of tumor tissues obtained from nude mice from MOCK or PGAM1 group. Scale bar = 100 μm (**j**). The results are presented as the mean ± SD for each group (*n* = 6). *p < 0.05, **p < 0.01 by Mann–Whitney *U*-test.

To further elucidate the oncogenic role of PGAM1 in vivo, stable PGAM1 knockdown or overexpression cells and control A549 cells were injected subcutaneously into BALB/c nude mice model (6 mice per group). The tumor volume and luciferase signal were measured on a weekly basis. After 5 weeks, we observed that the mice injected with PGAM1- knockdown A549 cells had reduced tumor weight and volume compared with those in mice injected with control cells (Fig. 4a–c). Consistently, the bioluminescence signal of tumors formed by the sh-PGAM1 cells was significantly decreased (Fig. 4d). Furthermore, PGAM1 and Ki-67 were significantly weaker in the PGAM1-silenced xenograft tumors than in control xenograft tumors (Fig. 4e). Conversely, as shown in Fig. 4f–j, tumors formed by PGAM1-overexpressing A549 cells were heavier and larger than tumors formed by control cells (Fig. 4f–i). Furthermore, IHC analysis revealed that PGAM1-overexpressing tumors showed higher percentages of Ki-67-positive cells (Fig. 4j). Collectively, our findings emphasize the role of oncogenic PGAM1 in NSCLC progression in vivo.

### PGAM1 overexpression promotes the TGF-β signaling pathway

**Fig. 5:**
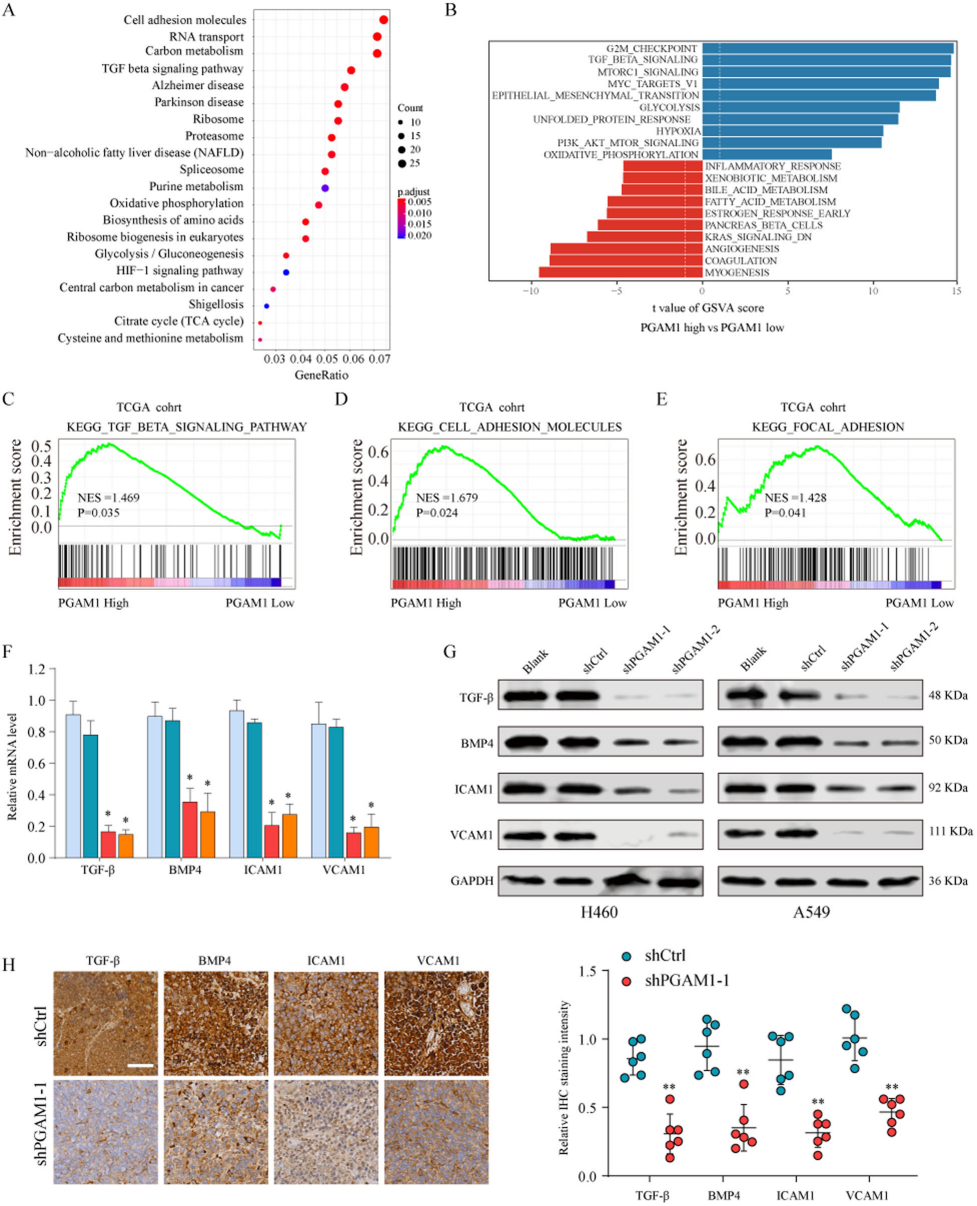
PGAM1 promotes NSCLC progression through activating TGF-β signaling pathway. Kyoto Encyclopedia of Genes and Genomes (KEGG) analysis (**a**) and Gene set variation analysis (GSVA) (**b**) were conducted in TCGA dataset to explore PGAM1-related downstream pathways. **c**–**e** GSEA analysis the enrichment of the TGF-β signaling pathway related genesets in NSCLC tissues with different PGAM1 expression level. Expression levels of TGF-β, BMP4, ICAM1 and VCAM1 in A549 cells transfected with shCtrl or shPGAM1 were analyzed by real-time PCR (**f**), western blot (**g**) in vitro. **h** The xenografts were embedded in paraffin blocks and paraffin sections were examined by IHC staining. Scale bars, 100 μm. *p < 0.05, **p < 0.01.

The underlying mechanism by which PGAM1 contributes to NSCLC progression remains unclear. We performed Kyoto Encyclopedia of Genes and Genomes (KEGG) analysis and gene set variation analysis (GSVA) to explore PGAM1-related downstream pathways in the TCGA NSCLC dataset. We found that the TGF-β signaling pathway was closely correlated with PGAM1 overexpression (Fig. 5a, b). We further confirmed the significant activation of the TGF-β signaling pathway, cell adhesion molecules and focal adhesion pathways in PGAM1-high-expression NSCLC tissues by gene set enrichment analysis (GSEA) analysis, indicating that the TGF-β signaling pathway may be involved in the oncogenic role of PGAM1 in NSCLC (Fig. 5c–e). To test this hypothesis, the expression of TGF-β signaling pathway related molecules (TGF-β, BMP4, ICAM1 and VCAM1) was determined through qRT-PCR and western blotting and the results showed that the expression levels of these molecules were significantly downregulated in PGAM1 silencing NSCLC cells (Fig. 5f, g). Consistently, IHC staining of xenograft tumor tissues using TGF-β, BMP4, ICAM1 and VCAM1 antibodies indicated suppression of the TGF-β signaling pathway status in PGAM1 silencing cancer cells compared to that in the control group (Fig. 5h). Moreover, it has been suggested that the TGF-β signaling pathway can induce the epithelial–mesenchymal transition (EMT) process in NSCLC cells^19^. Thus, we further investigated whether PGAM1 affects EMT in NSCLC cells. As expected, EMT-related gene sets were significantly enriched in the PGAM1-high expression phenotype, suggesting that PGAM1 may contribute to TGF-β-induced EMT of NSCLC cells (Supplementary Fig. S6A). Furthermore, PGAM1 silencing led to an increased E-cadherin expression and reduced N-cadherin, Snail and Slug expression in NCI-H226 and SK-MES-1 cells (Supplementary Fig. S6B, C). Collectively, our results reveal that PGAM1 activates the TGF-β signaling pathway and induces EMT during NSCLC tumorigenesis.

### PGAM1 is a direct target of miR-3614-5p

**Fig. 6:**
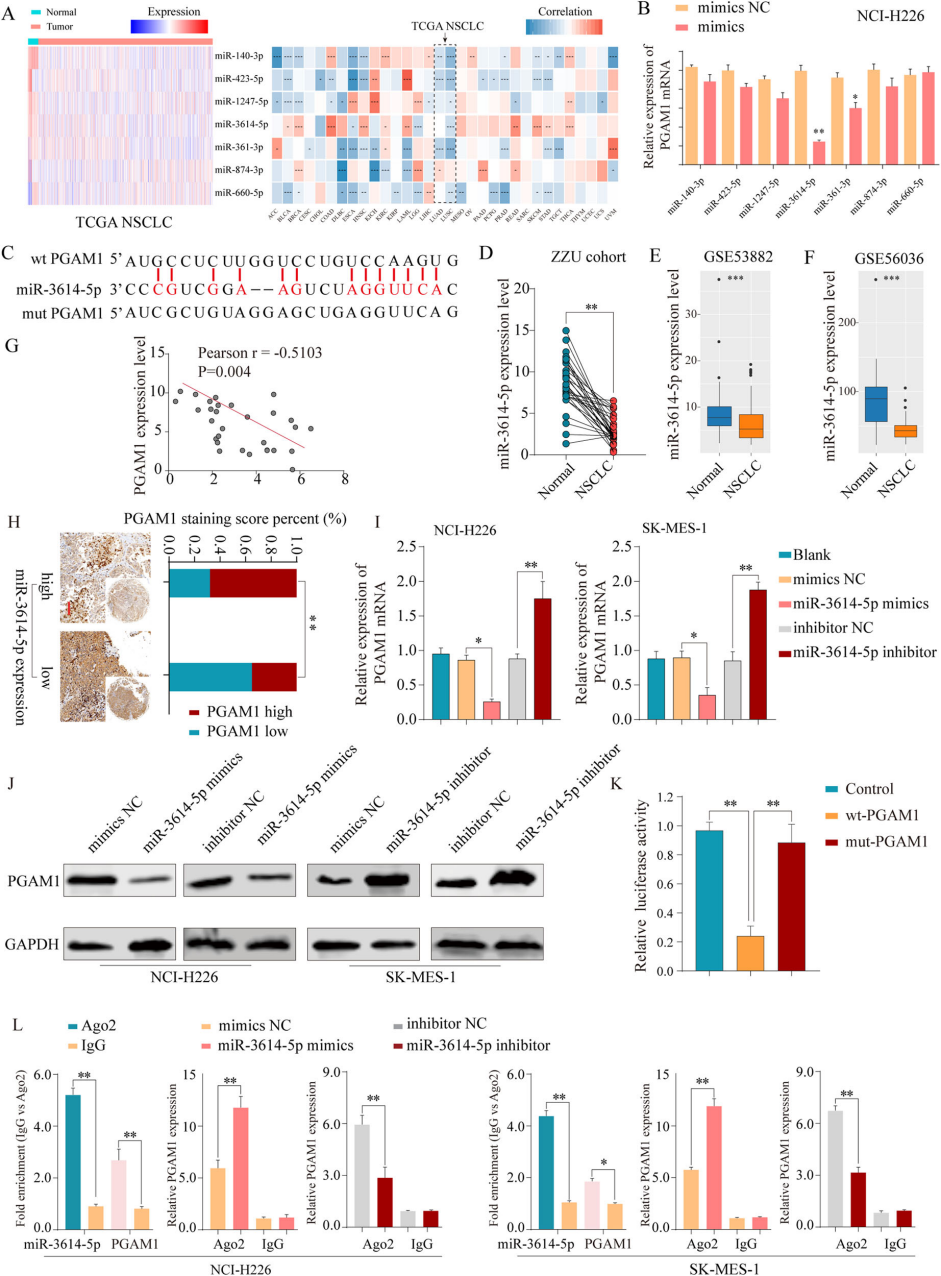
PGAM1 expression is downregulated by miR-3614-5p directly targeting of the 3’-UTR of PGAM1. **a** The expression status of candidate miRNAs targeting PGAM1 were analyzed based on TCGA dataset (left panel). Pearson analysis of the correlation between PGAM1 and candidate miRNAs based on TCGA database (right panel). **b** Expression of PGAM1 was detected under treatment of candidate miRNA mimics in NCI-H226 cells. **c** PGAM1 was found for the potential regulatory targets of miR-3614-5p using prediction tool (Starbase 3.0). The PGAM1 mRNA levels were determined by real-time PCR analysis in 30 paired NSCLC tissues (**d**), and in GSE53882 (**e**) and GSE56036 (**f**) datasets. **g** Pearson analysis of the correlation between PGAM1 and miR-3614-5p expression in 30 paired NSCLC specimens. **h** Representative IHC staining of PGAM1 and miR-3614-5p in NSCLC tissues (left panel) and quantification of PGAM1 staining scores in NSCLC patients from ZZU cohort with high or low miR-3614-5p expression (right panel). Scale bars, 100μm. The PGAM1 expression levels were determined by real-time PCR analysis (**i**) western blot (**j**) after transfection with the miR-3614-5p mimics or negative control or after transfection with the miR-3614-5p inhibitor or negative control in NCI-H226 and SK-MES-1 cells. GAPDH served as an internal control. **k** Luciferase activity assays for luciferase reporters with wild-type or mutant PGAM1 3’-UTR were performed after co-transfected with miR-3614-5p mimics or miR-control in 293 T cells. The luciferase activity of each sample was normalized to Renilla luciferase activity. **l** RIP assays confirmed the binding status between miR-3614-5p and PGAM1 in untreated and treated NSCLC cell lines, respectively. The representative result of at least three independent experiments was shown. *p < 0.05; **p < 0.01.

We next identified the molecular mechanisms responsible for PGAM1 overexpression in NSCLC. Numerous studies have identified that miRNAs exert critical roles in cancer progression by regulating target genes^14^. Seven candidate miRNAs targeting PGAM1 were selected based on an online prediction tool (StarBase 3.0) and expression status (Fig. 6a). Subsequently, we further performed validation experiments in vitro and found that miR-3614-5p expression significantly reduced PGAM1 mRNA levels (Fig. 6b). It has been reported that miR-3614-5p functions as a tumor suppressor in breast cancer^17^. We first confirmed the interaction between miR-3614-5p and PGAM1 with the sites predicted (Fig. 6c). We also found that miR-3614-5p expression was significantly decreased in NSCLC tissues (Fig. 6d–f, Supplementary Fig. S8A, B). Consistently, miR-3614-5p expression was inversely associated with mRNA levels (Fig. 6g) and strong IHC staining intensive of PGAM1 in NSCLC tissues (Fig. 6h). Moreover, we found that knockdown of miR-3614-5p significantly increased PGAM1 expression, whereas enforced expression of miR-3614-5p significantly decreased PGAM1 expression in NSCLC cells (Fig. 6i–j). Additionally, dual reporter luciferase assays indicated that the miR-3614-5p mimic significantly decreased the luciferase activity of PGAM1-wt but not PGAM1-mut (Fig. 6k). RIP assay results showed that miR-3614-5p and PGAM1 were both enriched in Ago2-coated beads relative to the IgG control group, further indicating the direct binding between miR-3614-5p and PGAM1 in NCI-H226 and SK-MES-1 cell lines (Fig. 6l). Additionally, given the previous reports that HIF1α could promote PGAM1 expression^11^, we further determined the potential effects of hypoxic condition on miR-3614-5p/PGAM1 axis. The results indicated that miR-3614-5p regulated the PGAM1 expression in a hypoxia-independent manner (Supplementary Fig. S7).

Moreover, although there was no significant relationship between miR-3614-5p expression level and NSCLC clinical outcome **(**Supplementary Fig. S8C, D), patients with a profile of miR-3614-5p^high^/PGAM1^low^ profiles had a better survival rate that patients with miR-3614-5p^low^/PGAM1^high^ profiles (Supplementary Fig. S8E, F). Overall, these results indicated that PGAM1 is a direct functional target of miR-3614-5p in NSCLC.

### The miR-3614-5p/PGAM1 axis promotes NSCLC progression via the TGF-β signaling pathway

**Fig. 7:**
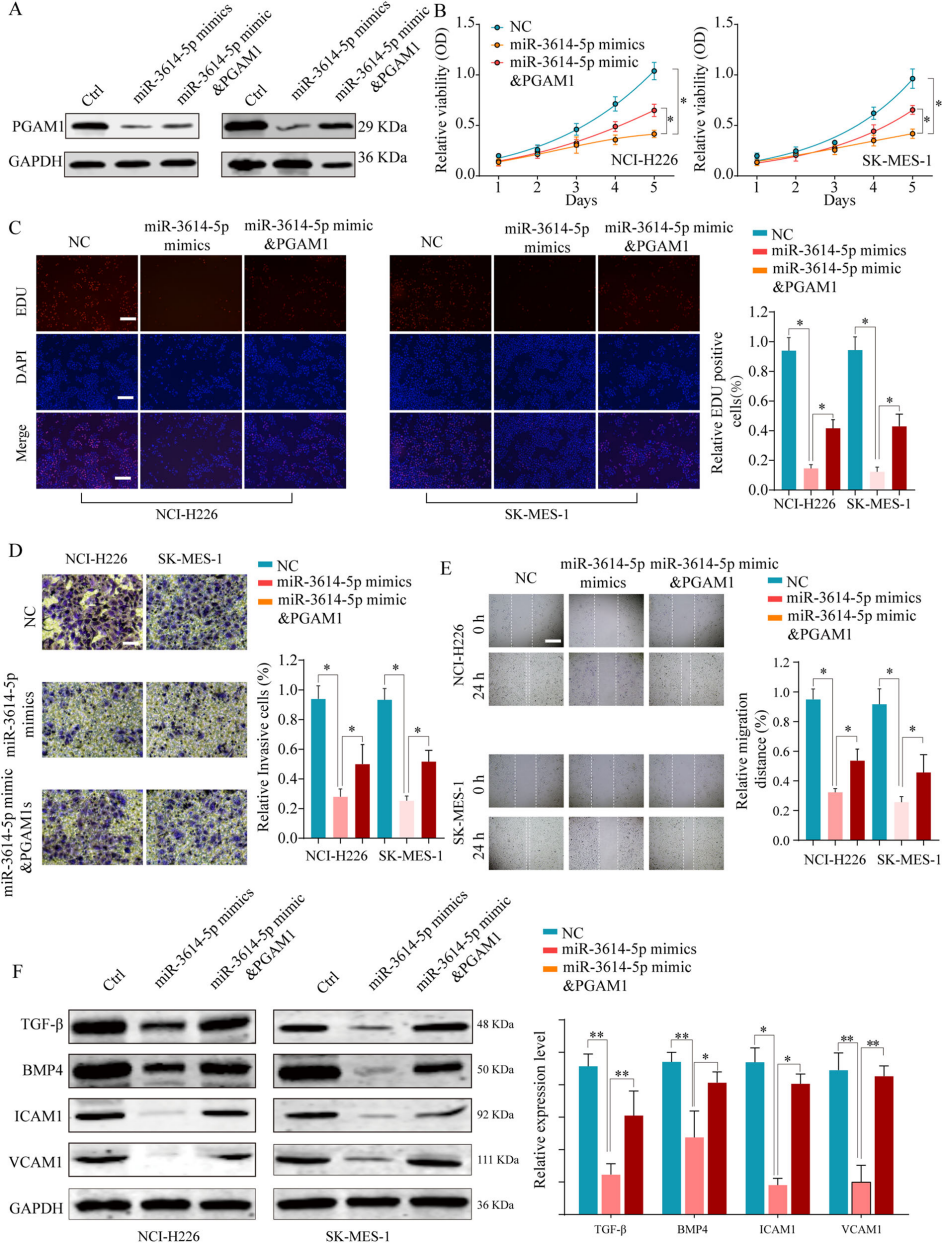
MiR-3614-5p inhibits NSCLC cell proliferation and invasion in vitro by inhibiting PGAM1. NSCLC cells NCI-H226 or SK-MES-1 were transfected with negative control (Ctrl), miR-3614-5p mimics, or miR-3614-5p mimics & PGAM1 overexpression plasmid. **a** The protein expression levels of PGAM1 in different groups were analyzed by western blot 48 h. **b** Cell viability was assessed at indicated time points by CCK-8 assay, **c** EDU incorporation, **d** transwell assays and **e** wound healing assays were performed to evaluate the NSCLC cell proliferation and invasion in vitro. **f** The protein expression of TGF-β, BMP4, ICAM1 and VCAM1 were analyzed by western blot. The representative result of at least three independent experiments was shown. Results were shown as mean ± SD. *p < 0.05, **p < 0.01.

Given the potential tumor suppressive role of miR-3614-5p in cancer, we further explored whether PGAM1 mediated the tumor suppressive role of miR-3614-5p in NSCLC. Ectopic expression of miR-3614-5p markedly suppressed PGAM1 expression, while co-transfection of miR-3614-5p with the PGAM1 overexpression plasmid mildly rescued the PGAM1 expression in NSCLC cell lines (Fig. 7a). Functional experiments showed that reintroduction of PGAM1 could partially reverse the inhibition of NSCLC cell proliferation ability caused by the miR-3614-5p overexpression (Fig. 7b, c). In addition, PGAM1 counteracted the decrease in cancer cell metastasis potential induced by ectopic expression of miR-3614-5p, as revealed by the transwell and migration assay (Fig. 7d, e). Furthermore, the expression of TGF-β signaling pathway related molecules was decreased after enforced expression of miR-3614-5p in NSCLC cells, while reintroduction of PGAM1 abolished the suppressive effect of miR-3614-5p mimics on the TGF-β signaling pathway (Fig. 7f).

Additionally, as PGAM1 is an important enzyme in the aerobic glycolysis process, we further confirmed the effect of miR-3614-5p/PGAM1 axis on NSCLC cell glucose metabolism (Supplementary Fig. S9)^12,20^. Taken together, these findings revealed that the miR-3614-5p/PGAM1 axis promotes NSCLC progression, at least in part, via the TGF-β signaling pathway.

### miR-3614-5p inhibits tumor growth in an NSCLC xenograft model

**Fig. 8:**
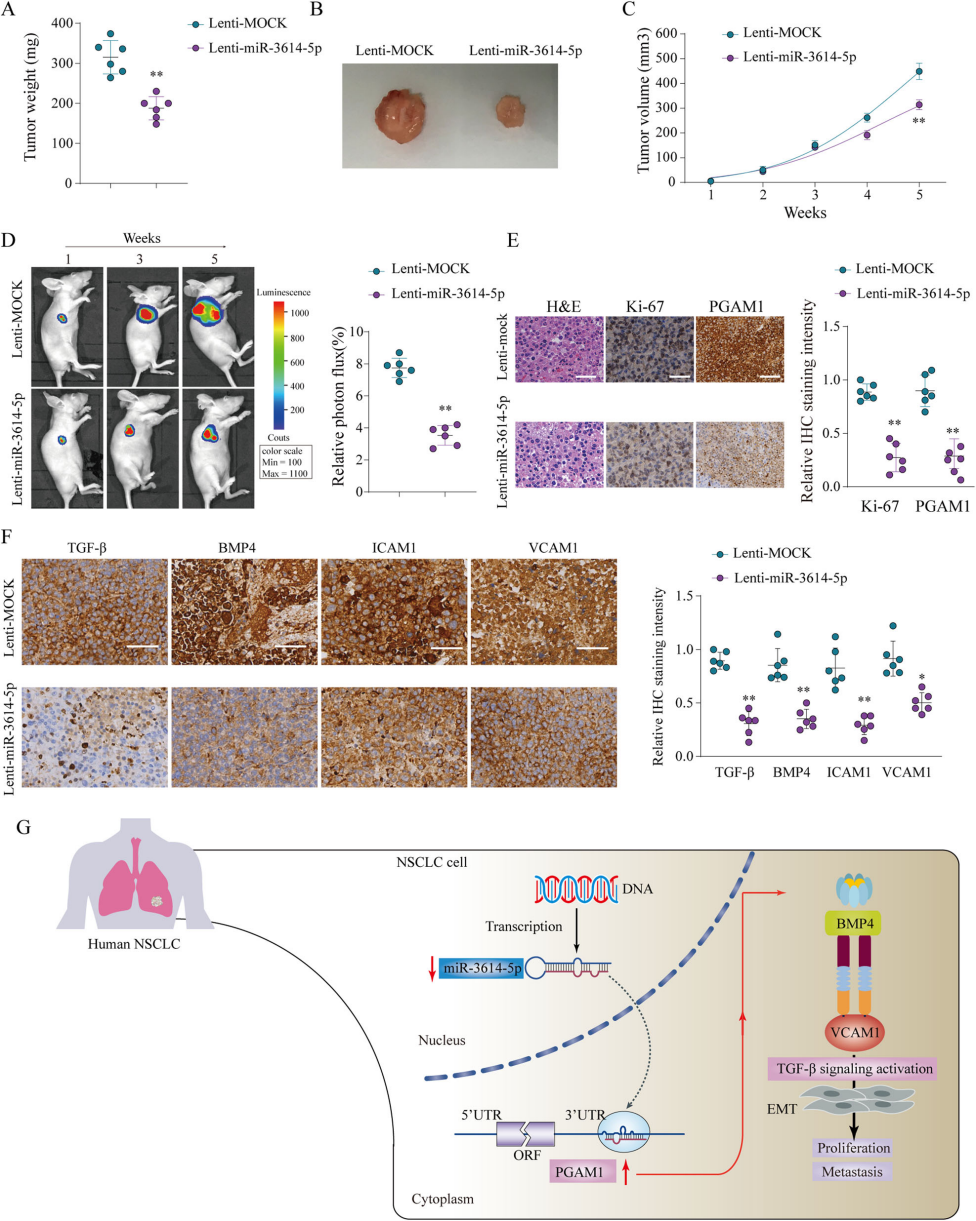
MiR-3614-5p suppress the proliferation of NSCLC cells in vivo. A549 cells infected by lentiviral to achieve miR-3614-5p stable overexpression (Lenti-miR-3614-5p) or infected by negative control (Lenti-MOCK) were implanted into the nude mice and tumor growth was recorded. Tumor weight in nude mice was assessed at week 5 (**a**, **b**) and tumor volume (**c**) was determined based on tumor size measured every week in nude mice from Lenti-miR-3614-5p or Lenti-MOCK group. Representative bioluminescent images and quantification of bioluminescent imaging signal intensities in nude mice from Lenti-miR-3614-5p or Lenti-MOCK group (**d**). Representative images of HE staining, Ki-67 and PGAM1 IHC staining of tumor tissues obtained from nude mice from Lenti-miR-3614-5p or Lenti-MOCK group. Scale bar = 100 μm (**e**). Expression levels of TGF-β, BMP4, ICAM1 and VCAM1 in A549 cells transfected with Lenti-miR-3614-5p or Lenti-MOCK were analyzed by IHC staining (**f**). A proposed regulated axis between miR-3614-5p/PGAM1 axis driving TGF-β-induced EMT in NSCLC progression (**g**). The results are presented as the mean ± SD for each group (*n* = 6). *p < 0.05, **p < 0.01 by Mann–Whitney *U*-test.

We further confirmed the suppressive role of miR-3614-5p in an NSCLC xenograft model. We subcutaneously inoculated A549 cells with overexpression of miR-3614-5p subcutaneously into nude mice. The results showed that mice injected with miR-3614-5p-overexpressing A549 cells (lenti-miR-3614-5p group) exhibited markedly reduced transplanted tumor weight and volume of transplanted tumors compared to that in the control group (lenti-MOCK) (Fig. 8a–c). Consistently, in vivo live imaging indicated that ectopic expression of miR-3614-5p significantly reduced photon flux (Fig. 8d). Additionally, IHC analysis demonstrated that PGAM1 and Ki-67 expression was significantly weaker in the lenti-miR-3614-5p group than in the lenti-MOCK group (Fig. 8e). Moreover, consistent with the results observed after PGAM1 knockdown, the TGF-β signaling pathway was significantly suppressed in mouse tumors formed by miR-3614-5p-overexpression cells (Fig. 8f). These results further demonstrate that miR-3614-5p expression suppresses NSCLC tumor growth by inhibiting PGAM1 through attenuating the TGFβ signaling pathway (Fig. 8g).

## Discussion

PGAM1 is a critical enzyme in coordinating glycolysis and biosynthesis^20^. Emerging evidence indicates that PGAM1 is frequently upregulated and promotes the progression of several cancers^5,7–9,11,21,22^. In NSCLC, Sun et al. revealed that PGAM1 was significantly upregulated and that elevated PGAM1 was positively correlated with poor survival^11^. Consistently, we determined the elevated expression of PGAM1 both at the mRNA and protein levels by combined analysis of publicly available datasets and our cohort, which was consistent with the results observed by proteomic analysis^23^. Additionally, PGAM1 in NSCLC tissues had a relatively high diagnostic efficacy. We also provide solid clinical evidence that PGAM1 overexpression in NSCLC correlated with poor prognosis in several independent NSCLC cohorts. Together, these findings and ours suggest that PGAM1 might serve as a potential diagnostic and prognostic biomarker for NSCLC patients.

Evidence has shown that repression of PGAM1 expression could inhibits proliferation, metastasis and chemotherapy resistance in multiple cancers^7,9,11,21^. For instance, PGAM1 promotes pancreatic ductal adenocarcinoma proliferation and metastasis^21^. In lung cancer, Sun et al. found that PGAM1 knockdown inhibited NSCLC xenografting tumorigenesis^11^. In agreement with these findings, we demonstrated that the PGAM1 promoted the malignant biological behavior of NSCLC cells. Additionally, decreased DNA synthesis and activation of the apoptosis pathway were observed after PGAM1 silencing. Based on these findings, we propose a potential therapeutic strategy for NSCLC that involves targeting PGAM1. KH3, an allosteric PGAM1 inhibitor, has shown desirable drug-like properties with satisfactory efficacy and limited toxicity in pancreatic ductal adenocarcinoma^12^. Interestingly another novel PGAM1 allosteric inhibitor, HKB99, has been reported to could suppress tumor growth and metastasis and overcome drug resistance in NSCLC^26^. Taken together, these findings strongly suggest that targeting PGAM1 is a potential strategy for treating NSCLC.

A previous study demonstrated that PGAM1, as a critical metabolic enzyme involved in glycolysis and biosynthesis, contributes to cancer progression by regulating cancer metabolism^11,20^. Consistently, we also confirmed that PGAM1 knockdown potently inhibited glucose metabolism. However, whether and how PGAM1 facilitates NSCLC progression through other mechanisms remains a subject for further study. To elucidate this issue, comprehensive bioinformatics analysis was performed, and the results indicated a significant positive correlation between elevated PGAM1 and activation of the TGF-β signaling pathway. Moreover, functional experiments confirmed that PGAM1 knockdown significantly suppressed the activation of the TGF-β signaling pathway. Emerging evidences has shown that TGF-β signaling participates in a diverse set of cellular processes, including cell proliferation, apoptosis and metastasis^24^. Previous publications also reported that TGF-β signaling plays a crucial carcinogenic role in NSCLC development by inducing EMT^19^. Consistently, our current study shows that PGAM1 augments EMT, which consequently strengthens the role of TGF-β signaling in the EMT process. These findings imply that PGAM1 could be involved in lung cancer tumorigenesis and progression, at least in part, by activating the TGF-β signaling pathway.

A large amount of evidence suggests that certain miRNAs participate in the cancer progression by targeting distinct mRNAs^13^. miRNAs, such as miR-421, miR-125b, miR-195, miR-374a and miR-216a, can function as either tumor suppressors or oncogenes in NSCLC progression^25–29^. In this study, we identified PGAM1 as a functional target of miR-3614-5p, and confirmed miR-3614-5p regulated PGAM1 in a hypoxia-independent manner. To date, there has few reports addressing miR-3614-5p in human cancers. Wang, Z. et al. reported that miR-3614-5p functions as a tumor suppressive role through targeting TRIM25 in breast cancer^17^. Shang, J et al. also showed that that miR-3614-5p may suppress WNT signal pathway through targeting NFATC2 in NSCLC cell^18^. Consistently, our functional experiments revealed that the tumor inhibition effect of miR-3614-5p is in part mediated by the downregulation of PGAM1 in vitro and in vivo. Furthermore, the suppressive effect of miR-3614-5p on the glucose metabolism and TGF-β signaling pathway was attenuated by ectopic expression of PGAM1. In addition, we found that NSCLC patients who with miR-3614-5p^low^/PGAM1^high^ profiles always had a poorer prognosis than those with miR-3614-5p^high^/PGAM1^low^ profiles. Together, these data identify miR-3614-5p as a novel determinant of PGAM1 expression and establish a novel miR-3614-5p/PGAM1/TGF-β signaling pathway for NSCLC progression.

## Conclusions

In conclusion, our findings further strengthened the conclusion that PGAM1 plays a critical oncogenic role in the progression of NSCLC. Mechanistic investigations suggested that PGAM1 was a functional target for miR-3614-5p. Furthermore, miR-3614-5p/PGAM1 axis regulated the malignant phenotype, at least in part, through activating the TGF-β signaling pathway in NSCLC. Overall, our data further emphasize the potential of miR-3614-5p/PGAM1 axis as a therapeutic target for NSCLC patients.

## Methods

### Expression data sets

Gene expression data for eight human lung cancer cohorts and corresponding clinical information were obtained from the Gene Expression Omnibus (GEO; GSE10072, GSE19188, GSE7670, GSE40791, GSE31320, GSE37745 and GSE42127) and The Cancer Genome Atlas (TCGA) database. For Kaplan-Meier analysis, patients were stratified into ‘low’ and ‘high’ expression based on auto-select best cutoff using R language (version 3.51), and the detailed codes were shown in Supplementary materials.

### Patient samples and TMA

Two independent cohorts containing paraffin embedding lung cancer specimens were used in present study: (1) TMA cohort containing a total of 94 NSCLC and adjacent normal tissues were purchased from Outdo Biotech (Shanghai, China) (Outdo cohort); (2) 85 pairs of NSCLC and adjacent normal tissues were collected from NSCLC patients who underwent surgical resection at the First Affiliated Hospital of Zhengzhou University from 2011 to December 2013 (ZZU cohort). The study was approved by the Institutional Review Board of the First Affiliated Hospital of Zhengzhou University. All patients provided written informed consent and the project was in accordance with the Helsinki Declaration of 1975.

### Cell lines and cell culture

Human bronchial epithelial cell 16HBE and NSCLC cell lines A549, H1299, NCI-H226, SK-MES-1 were purchased from Cell Bank of Chinese Academy of Sciences and maintained in RPMI 1640 (Gibco, USA) with 10% fetal bovine serum (Gibco, USA) at 37 °C in a humidified incubator with 5% CO_2_.

### Western blotting analysis

Cells were lysed with RIPA buffer (Beyotime, China) containing cocktail inhibitor (Roche, USA). The protein samples were resolved by SDS-PAGE and transferred onto PVDF membranes (Millipore, USA). The membranes were blocked and then incubated with primary antibodies overnight at 4 °C. Specific antibodies used in present study are listed in Supplementary Table 1. Subsequently, the membranes were incubated with corresponding secondary antibodies and visualized by ECL detection system as described in our previous study^34^.

### Luciferase activity assay and RNA immunoprecipitation (RIP)

The 3′-UTR fragment of PGAM1 containing the miR-3614-5p binding sequences were cloned into psiCHECK-2 vector (Promega, USA) with firefly luciferase reporter. Mutated plasmid was used as control. The cells were co-transfected with luciferase reporter construct, miR-3614-5p mimic or PGAM1 expression vector. Cells were collected after 24 h transfection and luciferase activity were measured using the Dual Luciferase Reporter Assay System (Promega, USA). The interaction between miR-3614-5p and PGAM1 was detected by performing RIP assay using anti- PGAM1 and the Magna RIP™ RNA-Binding Protein Immunoprecipitation Kit (Millipore, Bedford, MA) according to its instructions as previous described^30^.

### Immunohistochemical (IHC) staining

IHC was performed on 4 μm sections of paraffin-embedded tissues to determine the expression level of PGAM1 protein as previous described^31^. In brief, the TMA slides slides were incubated in PGAM1 antibody diluted 1:200 at 4 °C overnight and carried out using the EnVision™ system (DAKO, Demark). The images of TMA slides were obtained using a the NanoZoomer system (Hamamatsu Photonics Inc., Germany).

### Tumor formation assay in a nude mouse model

A549 cells were subcutaneously injected into either side of the flank area of 6-week-old nude mice (*n* = 6 mice per group). Tumor volumes in mice were calculated (1/2 × length × width^2^) on a weekly basis. After 5-week incubation, the mice were euthanized, and tumors were excised and subjected to IHC analysis. Tumor growth was monitored with tumor weight, and photographed by IVIS@ Lumina II system (Caliper Life Sciences, USA). Animal experiments were conducted in accordance with the Guide for the Care and Use of Laboratory Animals and were approved by the Animal Care and Use Committee of Zhengzhou University.

### RNA isolation and qRT-PCR analyses, knockdown and overexpression of PGAM1, oligonucleotides and transfection, cell proliferation assay, invasion and wound healing assay

These assays were described in the Supplementary Materials and Methods section.

### Statistical analysis

Results are presented as means ± SD. Statistical analysis was performed in GraphPad Prism, version 5.0 (GraphPad Prism Software, CA), using the Mann–Whitney test for comparison of two groups. The analysis of correlation between factors was performed by Pearson’s correlation coefficient rank test. Survival analyses were conducted using the Kaplan-Meier method, and the comparison was performed using the log-rank test. Cox proportional hazards regression models were adopted for the univariate and multivariate analyses. *p* < 0.05 was considered to indicate a significant difference.

## Supplementary information

Supplementary Figure Legends

Supplementary Figure S1

RSupplementary Figure S2

Supplementary Figure S3

Supplementary Figure S4

RSupplementary Figure S5

Supplementary Figure S6

Supplementary Figure S7

Supplementary Figure S8

Supplementary Figure S9

Supplementary materials

Supplementary Tables
