# Supplementary material for: PGAM1, regulated by miR-3614-5p, functions as an oncogene by activating transforming growth factor-β (TGF-β) signaling in the progression of non-small cell lung carcinoma
Source: Cell Death Dis. 2020 Aug 27;11(8):710. doi: 10.1038/s41419-020-02900-4 (PMC7453026; doi:10.1038/s41419-020-02900-4)
Supplement: Supplementary file 1 — Supplementary Figure Legends [file 41419_2020_2900_MOESM1_ESM.docx]

Supplementary Figures

Supplementary Figure S1. The organic speciality distribution of PGAM1 in various cancers. **(A)** Bioinformatics analysis of PGAM1 mRNA expression level in TCGA and GTEX Pan-cancer database. Abbreviation: Adrenocortical carcinoma (ACC), Bladder urothelial carcinoma (BLCA), Breast cancer (BRCA), Cholangiocarcinoma (CHOL), Colon cancer (COAD), DLBC (Lymphoid Neoplasm Diffuse Large B-cell Lymphoma), Esophagus cancer (ESCA), Glioblastoma multiforme (GBM), Head and Neck squamous cell carcinoma (HNSC), Kidney renal clear cell carcinoma (KIRC), Kidney renal papillary cell carcinoma (KIRP), Brain Lower Grade Glioma (LGG), Liver cancer (LIHC), Lung cancer (LUAD), Lung squamous cell carcinoma (LUSC), Ovarian serous cystadenocarcinoma (OV), Pancreatic adenocarcinoma (PAAD), Pheochromocytoma and Paraganglioma (PCPG), Prostate adenocarcinoma (PRAD), Rectal cancer (READ), Sarcoma (SARC), Skin Cutaneous Melanoma (SKCM), Stomach cancer (STAD), Testicular Germ Cell Tumors (TGCT), Thyroid cancer (THCA), Thymoma (THYM) and Uterine Carcinosarcoma (UCS), Acute Myeloid Leukemia (LAML). **(B)** Differential expression of PGAM1 and PGAM2 in NSCLC tissues and normal tissues. **(C)** The protein expression levels of PGAM2 in bronchial epithelial cell 16HBE and NSCLC cell lines (A549, H1299, NCI-H226 and SK-MES-1) were analyzed by western blot. **(D)** The PGAM2 expression in rat heart muscle were analyzed by IHC. **(E)** Two paraffin sections obtained from a tumor tissues of NSCLC patient were examined by IHC staining using PGAM1 and PGAM2 antibody. **p < 0.01, ***p < 0.01.

Figure S2. Correlation between PGAM1 and Ki-67 or PCNA mRNA levels in normal tissues or NSCLC tissues. Relationship between expression level of PGAM1 and Ki67 or PCNA in TCGA dataset. Pearson’s correlation coefficient test was used.

Figure S3. ROC curve analysis of PGAM1 for the diagnosis of NSCLC tissues from normal tissues. AUC of PGAM1 mRNA expression for discriminating NSCLC tissues from normal tissues in TCGA dataset (A), GSE19188 dataset (B) GSE7670 dataset (C) and GSE10072 dataset (D).

Figure S4 PGAM1 promotes cell apoptosis of NSCLC cells in vitro

(A) Cell apoptosis of NCI-H226 and SK-MES-1 cells transfected with shCtrl or shPGAM1-1/2 lentivirus were analyzed by TUNEL staining. Expression levels of invasion related proteins MMP-2, MMP-7, MMP-9 (B) and apoptosis related proteins BCL2, BAX, BAK and cytochrome C (C) were analyzed by western blot. The representative result of at least three independent experiments was shown. Results were shown as mean ± SD. ***p* < 0.01.

**Figure S5 Overexpression of PGAM1 promotes cell proliferation, migration and invasion of NSCLC cells *in vitro*.
(A)** qPCR and Western blot analysis of PGAM1 expression in H460 or A549 cells after transfection of PGAM1 overexpression plasmid (PGAM1) or negative control (vector). Cell proliferation were analyzed by CCK-8 assay (**C**), colony formation assay (**D**) and EdU immunofluorescence staining (**E**) respectively. **(E)** Cell invasion ability was determined by transwell assay. **(F)** Cell invasion capability was assessed by transwell assay. The representative result of at least three independent experiments was shown. Results were shown as mean ± SD. **p* < 0.05, ***p* < 0.01

**Figure S6 PGAM1 promotes epithelial-to-mesenchymal transition (EMT) process in NSCLC cells.**

(**A**) The Gene Set Enrichment Analysis (GSEA) of the relationship between the expression level of PGAM1 and EMT-related gene signatures in TCGA cohort. (**B**) A mesenchymal marker, N-cadherin, E-cadherin and snail were downregulated in PGAM1 silencing NSCLC cells. Results were shown as mean ± SD. **p* < 0.05, ***p* < 0.01

**Figure S7. The miR-3614-5p regulated PGAM1 in a hypoxia-independent manner.**

NCI-H226 and SK-MES-1 underwent hypoxic chamber stimulation for 24 hours. **(A)** The protein expression of HIF1α, PGAM1, VEGF and SNAIL were analyzed by western blot. **(B)** The expression of miR-3614-5p was analyzed by RT-qPCR. Results were shown as mean ± SD.

**Figure S8.** **MiR-3614-5p/PGAM1 axis correlates with NSCLC patient outcomes**. (**A, B**) MiR-3614-5p expression levels in NSCLC tissues or non-tumor control tissues were analyzed in TCGA LUAD and LUSC cohorts. (**C-D**) Kaplan-Meier analysis of the overall survival in TCGA LUAD and LUSC cohorts according to the expression level of MiR-3614-5p. (**E-F**) Kaplan-Meier analysis of the overall survival in TCGA LUAD and LUSC cohorts according to the concurrent expression of MiR-3614-5p and PGAM1.

**Figure S9. The miR-3614-5p/PGAM1 axis regulated glucose metabolism**

NSCLC cells NCI-H226 and SK-MES-1 were transfected with negative control (NC), miR-3614-5p mimics, siRNA targting PGAM1 (PGAM1) or miR-3614-5p mimics & PGAM1 overexpression plasmid (PGAM1). Glucose consumption **(A)** and Lactate production **(B)** were measured. Results were shown as mean ± SD. **p* < 0.05, ***p* < 0.01
