# Supplementary figures and images for: PGAM1, regulated by miR-3614-5p, functions as an oncogene by activating transforming growth factor-β (TGF-β) signaling in the progression of non-small cell lung carcinoma
Source: Cell Death Dis. 2020 Aug 27;11(8):710. doi: 10.1038/s41419-020-02900-4 (PMC7453026; doi:10.1038/s41419-020-02900-4)

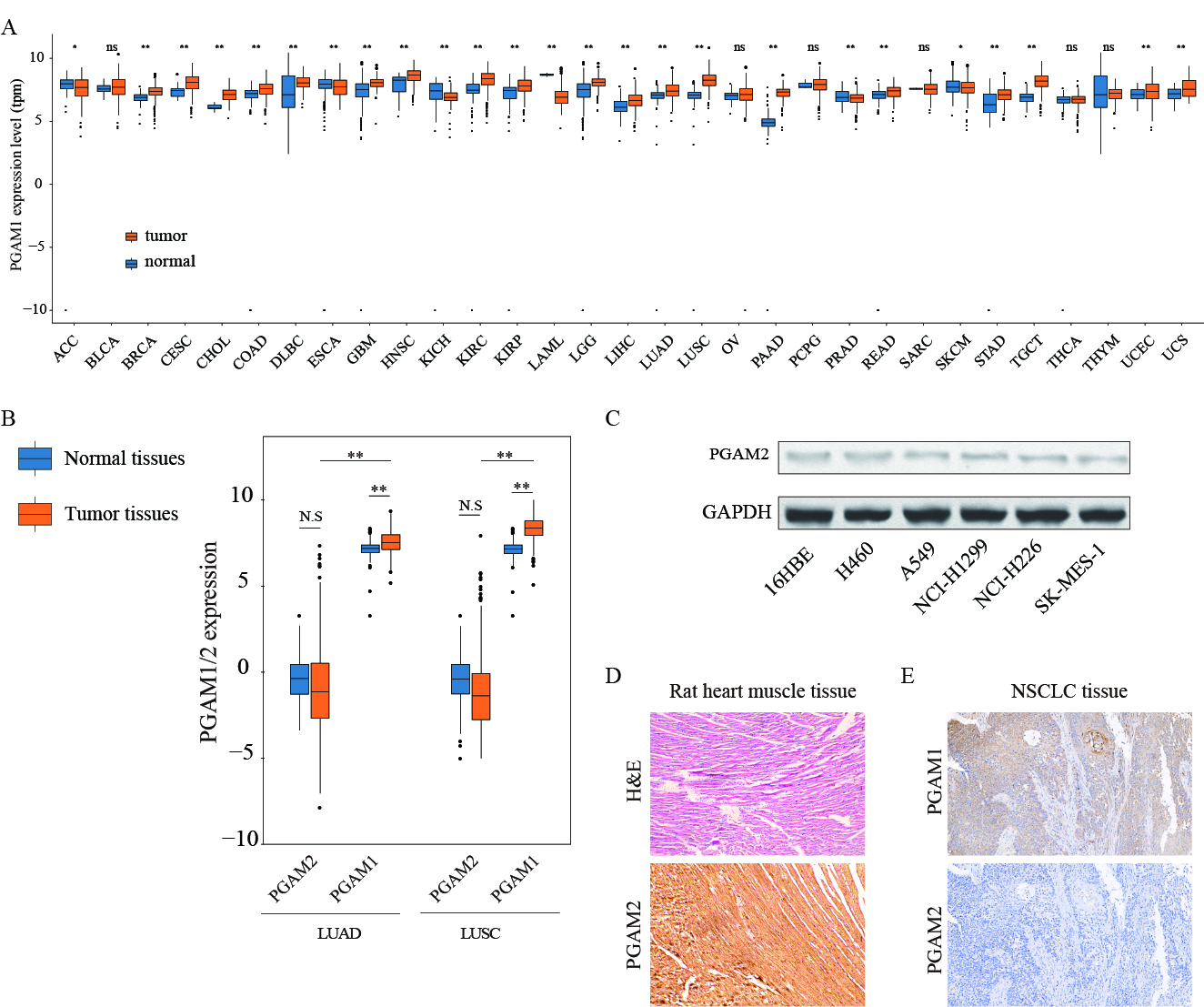

Supplement: Supplementary file 2 — Supplementary Figure S1 [file 41419_2020_2900_MOESM2_ESM.tif]

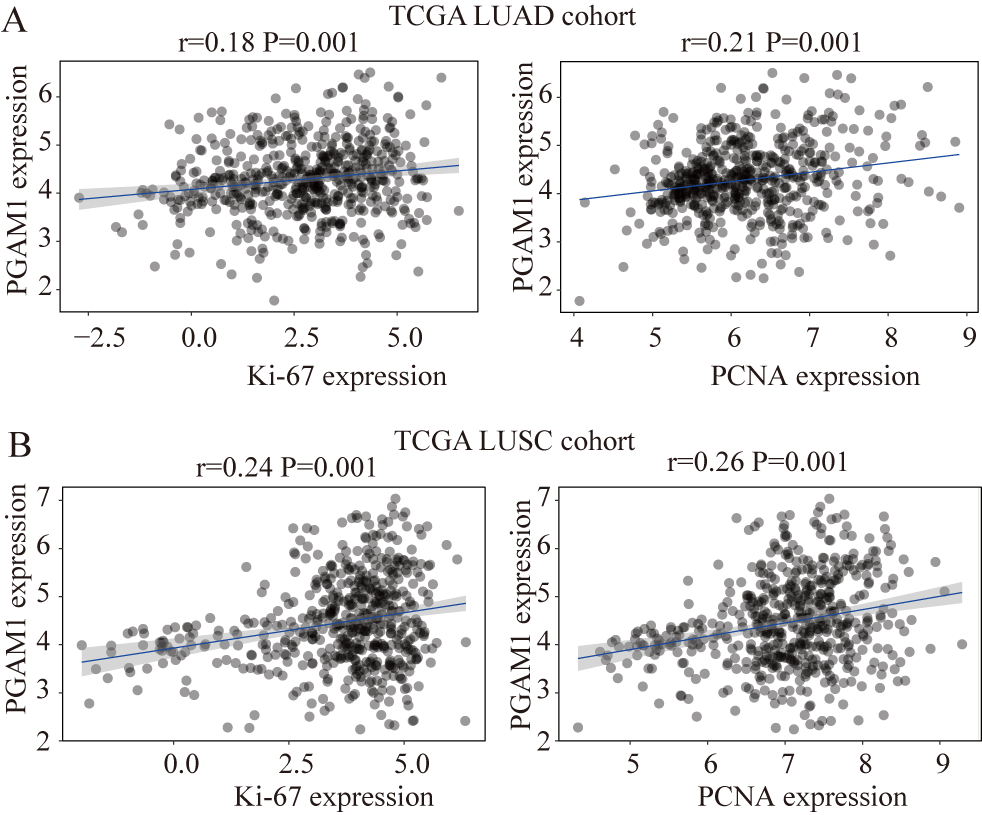

Supplement: Supplementary file 3 — RSupplementary Figure S2 [file 41419_2020_2900_MOESM3_ESM.tif]

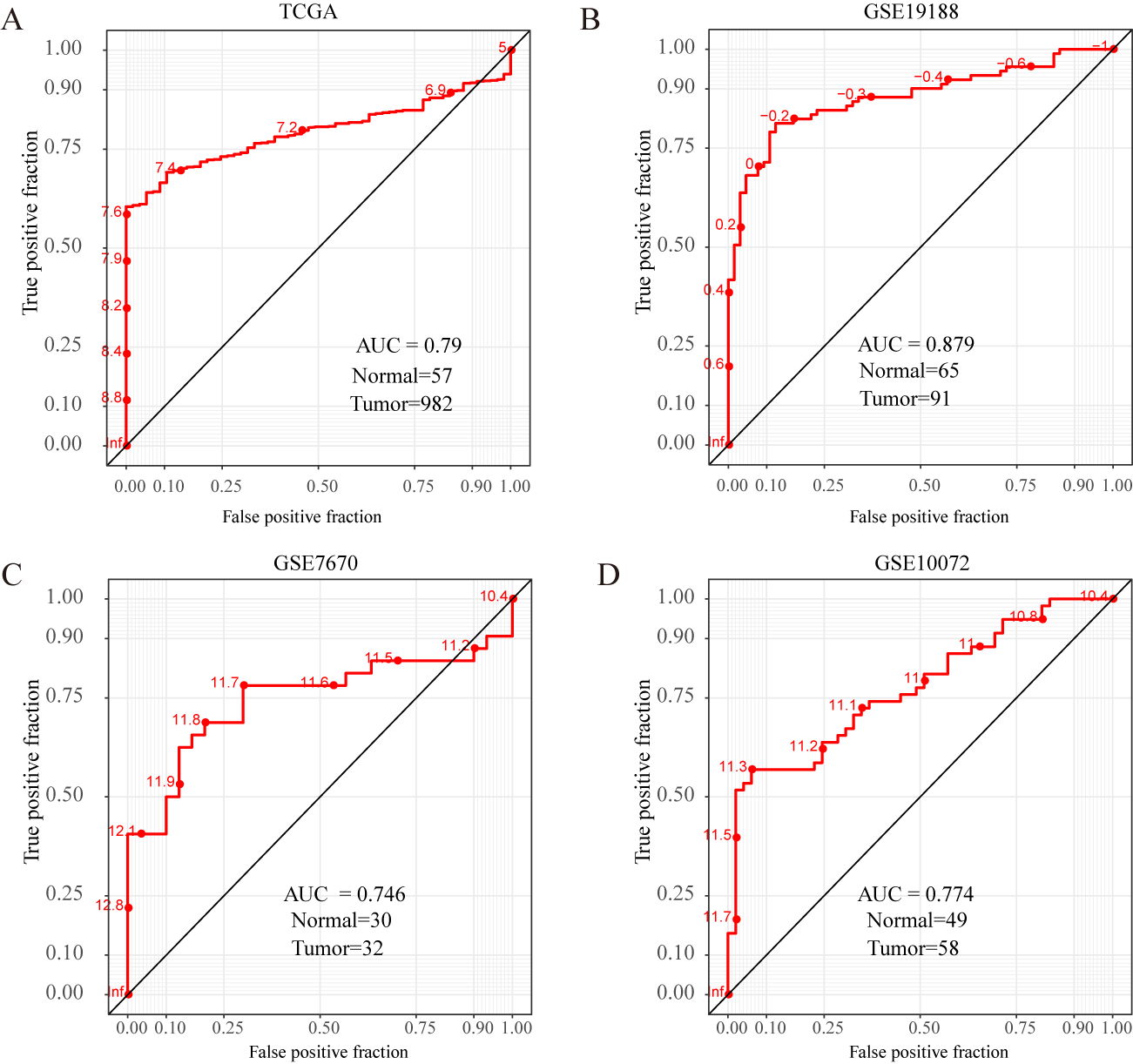

Supplement: Supplementary file 4 — Supplementary Figure S3 [file 41419_2020_2900_MOESM4_ESM.tif]

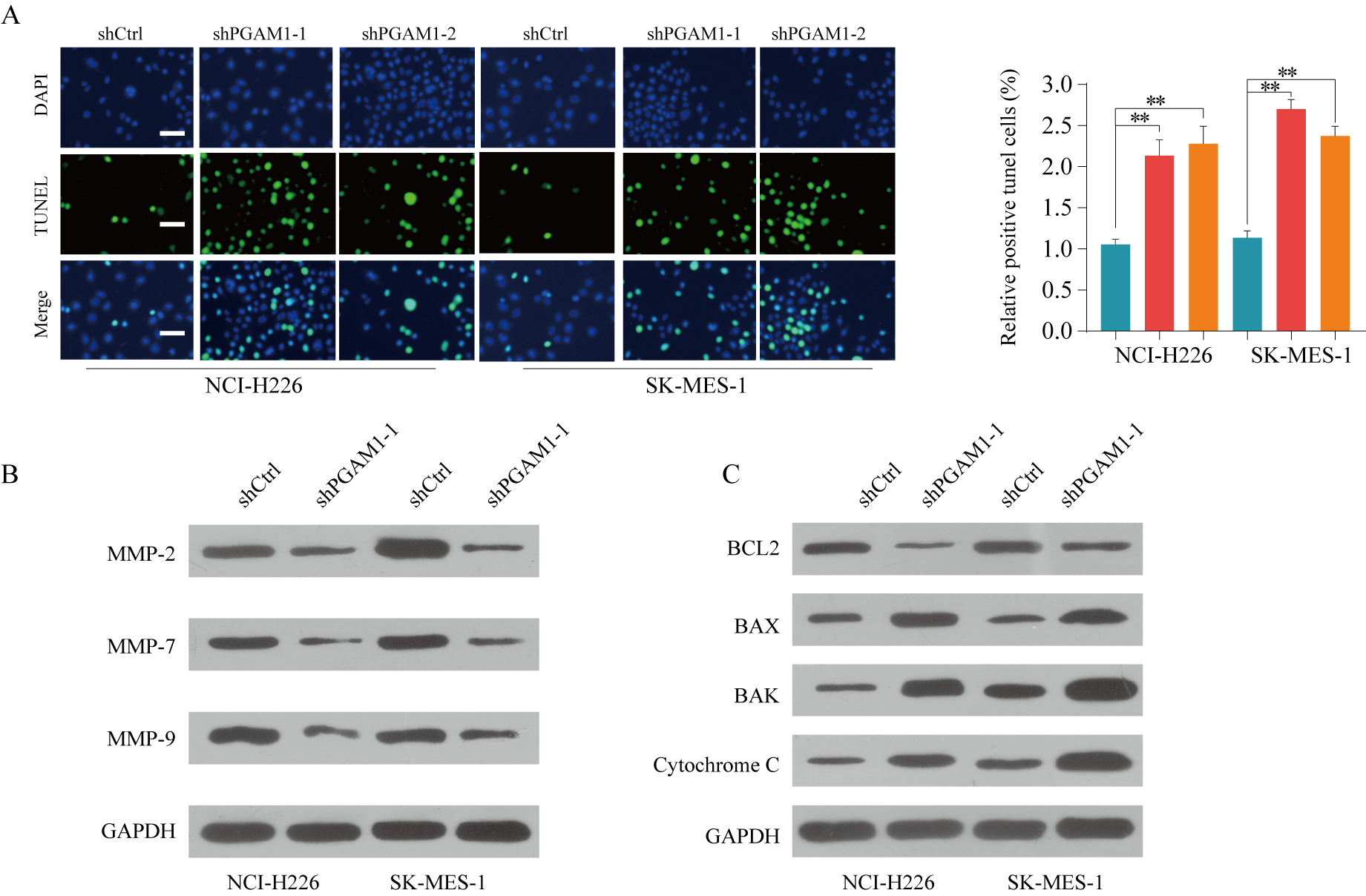

Supplement: Supplementary file 5 — Supplementary Figure S4 [file 41419_2020_2900_MOESM5_ESM.tif]

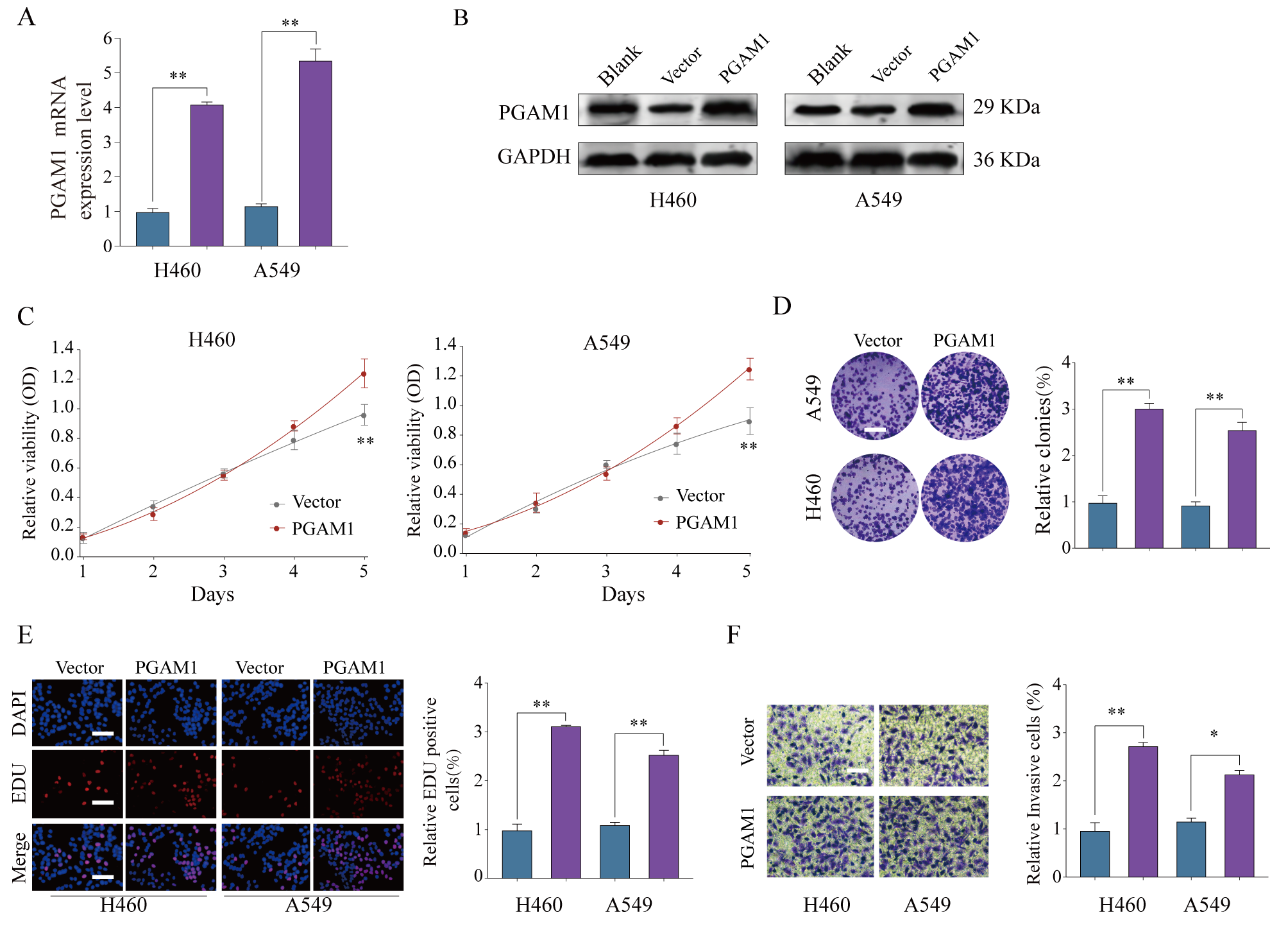

Supplement: Supplementary file 6 — RSupplementary Figure S5 [file 41419_2020_2900_MOESM6_ESM.tif]

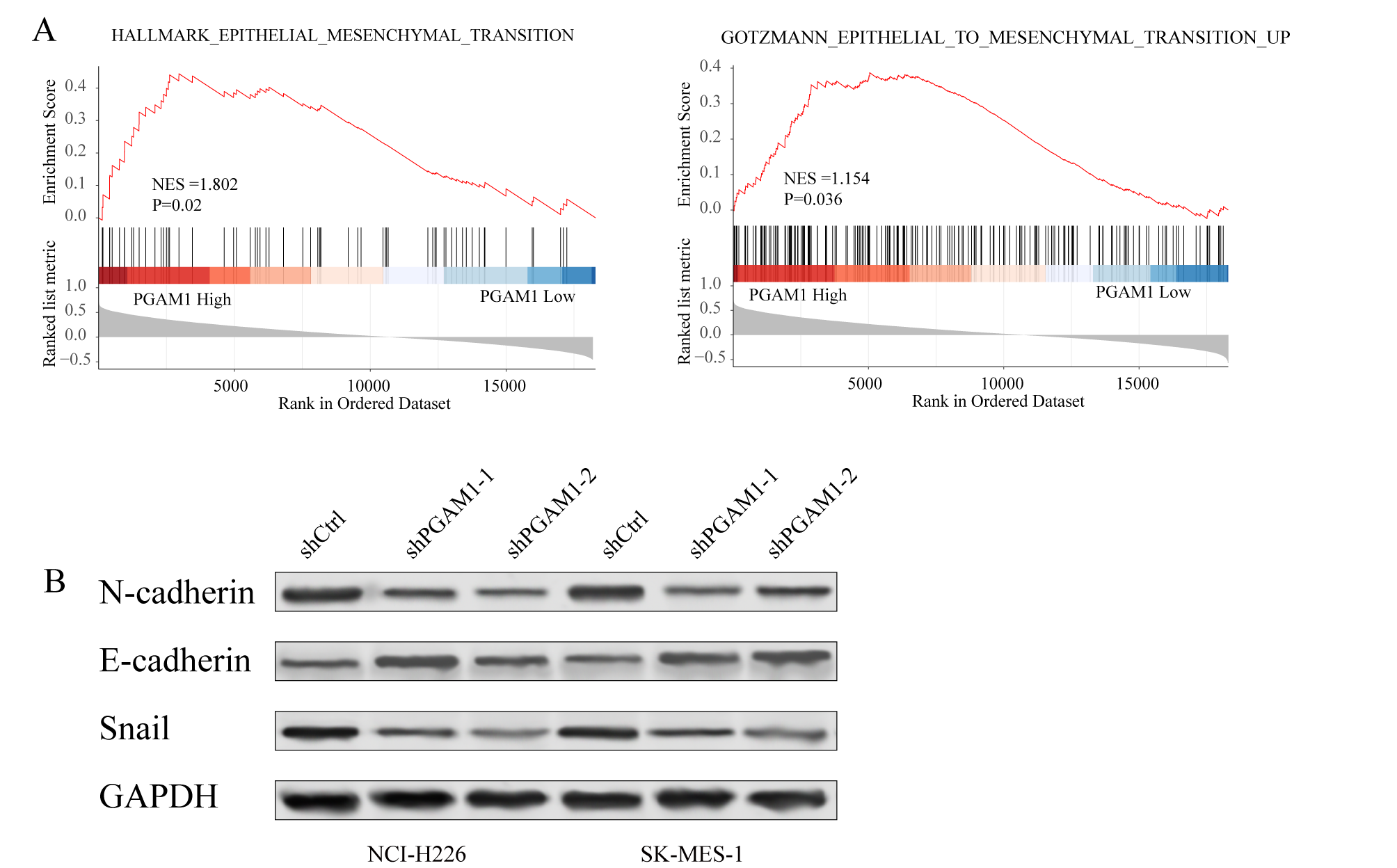

Supplement: Supplementary file 7 — Supplementary Figure S6 [file 41419_2020_2900_MOESM7_ESM.tif]

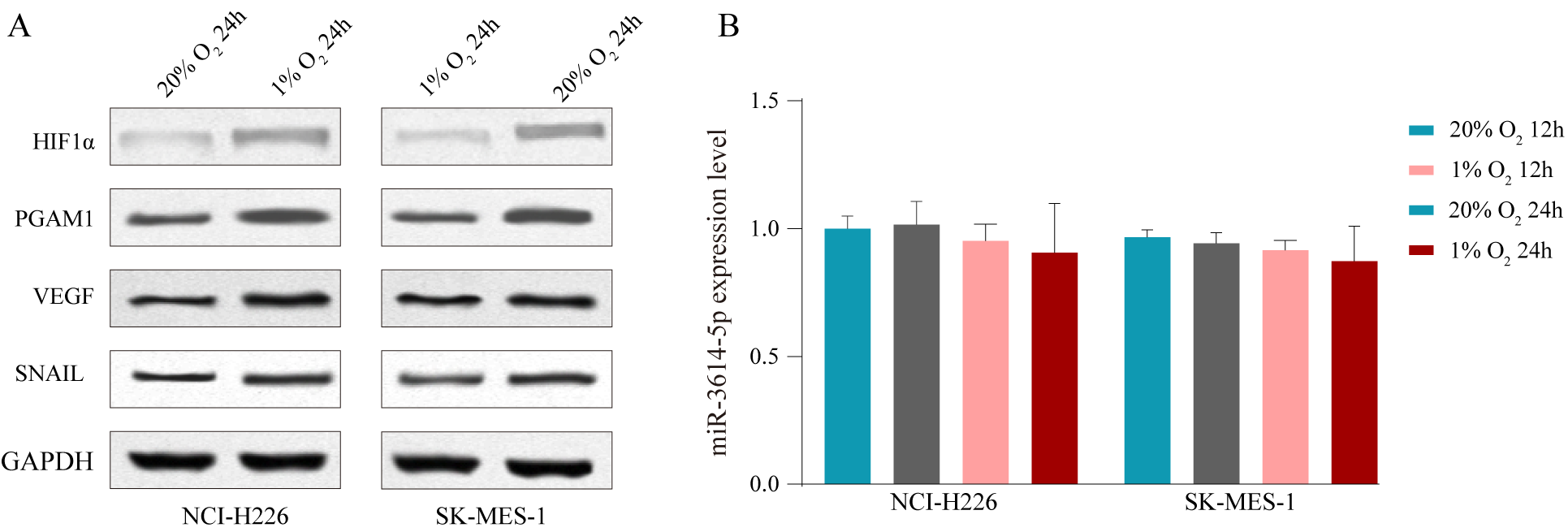

Supplement: Supplementary file 8 — Supplementary Figure S7 [file 41419_2020_2900_MOESM8_ESM.tif]

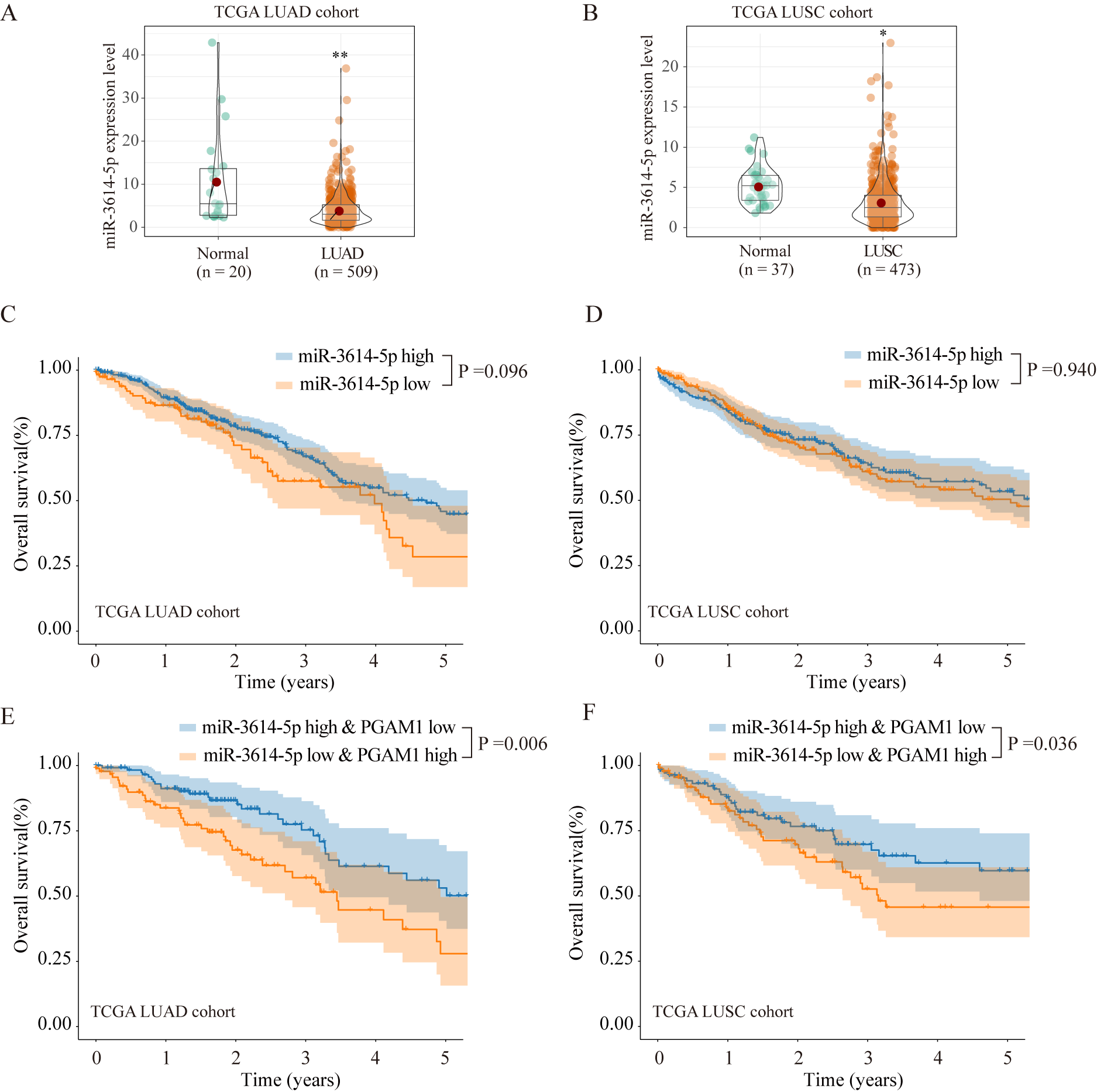

Supplement: Supplementary file 9 — Supplementary Figure S8 [file 41419_2020_2900_MOESM9_ESM.tif]

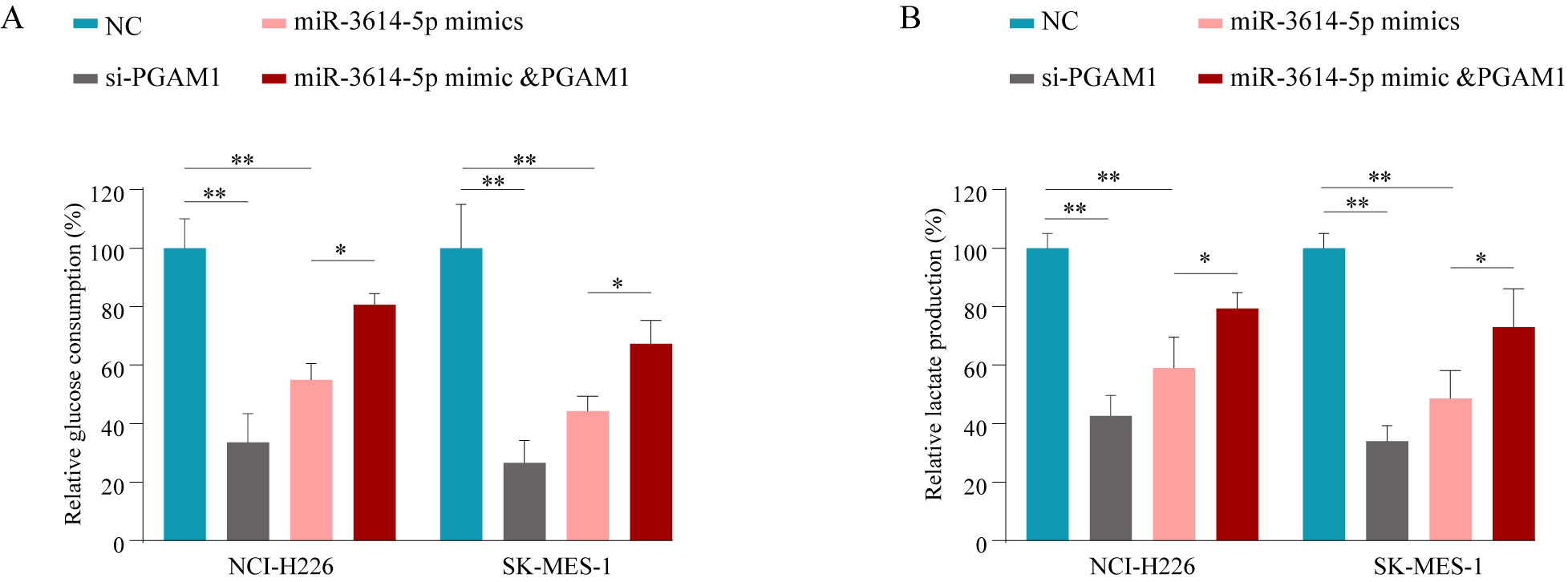

Supplement: Supplementary file 10 — Supplementary Figure S9 [file 41419_2020_2900_MOESM10_ESM.tif]
