# Supplementary material for: PGAM1, regulated by miR-3614-5p, functions as an oncogene by activating transforming growth factor-β (TGF-β) signaling in the progression of non-small cell lung carcinoma
Source: Cell Death Dis. 2020 Aug 27;11(8):710. doi: 10.1038/s41419-020-02900-4 (PMC7453026; doi:10.1038/s41419-020-02900-4)
Supplement: Supplementary file 11 — Supplementary materials [file 41419_2020_2900_MOESM11_ESM.docx]

**Supplementary materials**

**R code of selecting best cutoff for Kaplan-Meier survival analysis**

library(cutoff)

library(survminer)

library(survival)

dat$expression_median<-ifelse(dat$mygene>median(dat$mygene),'high','low')

table(dat$expression_median)

oscutoff<-logrank(data=dat,

time = 'ostime',y='os', x='mygene',

cut.numb=1,

n.per=0.25,

y.per=0.00,

p.cut=0.7)

oscutoff<-oscutoff[order(oscutoff$pvalue),]

oscutoff<-oscutoff$cut1[1]

dat$expression_bestcutoff<-ifelse(dat$mygene>oscutoff,'high','low')

table(dat$expression_bestcutoff)

fitOS_bestcutoff<- survfit(Surv(ostime, os) ~ expression_bestcutoff, data = dat)

ggsurvplot(

fitOS_median,

risk.table = FALSE,

conf.int = FALSE,

pval = TRUE,

xlab ='Time (days)',

break.time.by = 365,

risk.table.y.text.col = T,

legend = c(0.8, 0.9),

legend.title = "", #change legend title

palette = c('d3'),

main = "Survival curves",

risk.table.title = "Number at risk ",

risk.table.height= 0.25

)

**RNA isolation and qRT-PCR analyses**

Total RNA was extracted from the cultured cells or tissues using TRIZOL reagent (TAKARA, Japan) according to the manufacturer’s instructions. qRT-PCR analyses were conducted with SYBR Premix Ex TaqTM (Tli RNaseH Plus) (TAKARA, Japan) according to the manufacturer’s instructions. The primers sequences used in this study were listed in **Supplementary Table 2**. ABI Step One Plus was used to perform the amplification reaction. The results are presented as the mean ±standard deviation (SD) for duplicate runs. The relative quantification of PGAM1 expression was calculated using the 2^−△△CT^ method relative to GAPDH.

**Knockdown and overexpression of** **PGAM1**

Lentivirus expressing scramble or PGAM1 shRNAs was purchased from Hanbio Company (Shanghai, China). RNAi sequence used in this study were listed in **Supplementary Table 3.** In the case of knockdown experiments, cells were infected these lentiviral particles and selected with 3 μg/ml puromycin. In the case of overexpression experiments, cells were infected with lentiviral particles expressing empty vector control or PGAM1 (Hanbio Company) and selected with 3 μg/ml puromycin.

**Oligonucleotides and transfection**

The miR-3614-5p mimic and miR-3614-5p inhibitor, and their negative controls (NC mimic and NC inhibitor, respectively) were purchased from Ribobio (Guangzhou, China). The siRNAs specifically targeting PGAM1 (si-PGAM1) and scrambled control siRNA (si-NC) were synthesized by GenePharma (Shanghai, China). All cell transfection procedures were performed with Lipofectamine 2000 (Life Technologies).

**Cell proliferation assay**

The cell proliferation was measured by the Cell Counting Kit-8 (CCK-8) assays. The cells were seeded in a 96-well plate at a density of 1500 cells/well, and 10 μl of CCK-8 was added to 90 μl of the culture medium per well. The cells were next incubated for 2 h, and cell viability was measured as absorbance at 450 nm. For colony formation assay, 1000 cells were seeded in each well of a 6-well plate and incubated at 37°C for 2 weeks. The colonies were fixed and stained with a solution containing 0.1% of crystal violet and 20% of methanol and were the counted. All assays were performed three times. The DNA synthesized rate was determined with EdU assay kit (Ribobio, Guangzhou, China) according to the manufacturer’s instructions.

**Invasion and Wound healing assay**

For the invasion assays, 4×10^4^ cells were placed into the upper chamber with a Matrigel-coated membrane. After several hours of incubation at 37°C, cells that migrated or invaded were fixed with 20% methanol and stained with a dye solution containing 0.1% crystal violet. For the migration assays, 1×10^5^ cells were placed into the top chamber (BD Biosciences, NJ), and 500 μL medium with 10% FBS serum into under chamber. After 24h incubation, the invaded cells on the lower surface were fixed and stained. Finally, invaded cells were calculated under a microscope (Olympus, Japan). The cells that migrated or invaded were visualized and photographed using an inverted microscope (Olympus, Tokyo, Japan). Experiments were independently repeated three times.
