# Supplementary material for: PGAM1, regulated by miR-3614-5p, functions as an oncogene by activating transforming growth factor-β (TGF-β) signaling in the progression of non-small cell lung carcinoma
Source: Cell Death Dis. 2020 Aug 27;11(8):710. doi: 10.1038/s41419-020-02900-4 (PMC7453026; doi:10.1038/s41419-020-02900-4)
Supplement: Supplementary file 12 — Supplementary Tables [file 41419_2020_2900_MOESM12_ESM.docx]

**Supplementary Table 1. Information on antibodies used in this study**

| **Antibody** | **WB** | **IHC** | **Specificity** | **Company** |
| --- | --- | --- | --- | --- |
| PGAM1 | 1:2000 | 1:100 | Mouse monoclonal | ab129191, Aacam, USA |
| Ki-67 | / | 1:500 | Rabbit Polyclonal | Proteintech Group, China |
| GAPDH | 1:1000 | / | Mouse monoclonal | ab181602, Abcam, USA |
| TGF-β | 1:1000 | 1:600/ | Rabbit Polyclonal | ab31013, Abcam, USA |
| BMP4 | 1:1000 | 1:800/ | Rabbit Polyclonal | ab39973, Abcam, USA |
| ICAM1 | 1:5000 | 1:1500/ | Rabbit Polyclonal | ab221777, Abcam, USA |
| VCAM1 | 1:1000 | 1:1000/ | Rabbit monoclonal | ab134047, Abcam, USA |
| \| MMP-2 \| 1:1000 \| / \| Rabbit Polyclonal \| 10373-1-AP, Proteintech Group, China \| \| --- \| --- \| --- \| --- \| --- \| \| MMP-7 \| 1:1000 \| / \| Rabbit Polyclonal \| 10374-1-AP, Proteintech Group, China \| \| MMP-9 \| 1:5000 \| / \| Rabbit Polyclonal \| 10375-1-AP, Proteintech Group, China \|   MMP-2 | 1:2000 | / | Rabbit Polyclonal | 25614-1-AP,Proteintech Group , China |
| MMP-7 | 1:5000 | / | Rabbit Polyclonal | 10508-1-AP,Proteintech Group , China |
| MMP-9 | 1:2000 | / | Rabbit Polyclonal | 10828-1-AP,Proteintech Group , China |
| Bcl-2 | 1:1000 | / | Rabbit Polyclonal | 12789-1-AP,Proteintech Group , China |
| Bax | 1:4000 | / | Rabbit Polyclonal | 50599-2-Ig,Proteintech Group , China |
| Bak | 1:1000 | / | Rabbit monoclonal | D4E4, Cell Signaling, USA |
| Cytochrome C | 1:5000 | / | Mouse monoclonal | 66264-2-Ig,Proteintech Group , China |

**Supplementary Table 2. Primer sequence used in this study**

| **siRNA** | **sense sequence** |
| --- | --- |
| miR-3614-5p forward primer | 5'- AACAAGCCACTTGGATCTGAAGG -3' |
| miR-3614-5p reverse primer | 5'- CAGTGCAGGGTCCGAGGT -3' |
| U6 forward primer | 5'-CCAGUUUACCUAACGCAAUTT-3' |
| U6 reverse primer | 5'-TTCACGAATTTGCGTGTCAT-3' |
| GAPDH forward primer | 5′-ACCAGGAAATGAGCTTGACA-3′ |
| GAPDH reverse primer | 5′-GACCACAGTCCATGCCATC-3′ |
| PGAM1 forward primer | 5′- GTGCAGAAGAGAGCGATCCG -3′ |
| PGAM1 reverse primer | 5′- CGGTTAGACCCCCATAGTGC -3′ |

**Supplementary Table 3.** **RNAi sequence used in this study**

| **siRNA** | **sense sequence** |
| --- | --- |
| miR-3614-5p mimics | 5'- CCACUUGGAUCUGAAGGCUGCCC -3' |
| miR-3614-5p mimics negative  control | 5’-UCACAACCUCCUAGAAAGAGUAGA-3' |
| miR-3614-5p inhibitor | 5'- GGGCAGCCTTCAGATCCAAGTGG -3' |
| miR-3614-5p inhibitor negative control | 5'-TCTACTCTTTCTAGGAGGTTGTGA-3' |
| PGAM1 -shRNA-1 | 5'- CACCGCACAGGTATTTGGCCTCAGACGAATCTGAGGCCAAATACCTGTGC -3' |
| PGAM1 -shRNA-2 | 5'- CACCAGGTATTTGGCCTCAGATTGCCGAAGCAATCTGAGGCCAAATACC -3' |
| PGAM1 -shRNA-3 | 5'- CACCGCTTCTTGCCTTCACTGTACCCGAAGGTACAGTGAAGGCAAGAAGC -3' |
| Negative control shRNA | 5'-CACCCGACGTGCACCACGTGCTACTCGTACTCTTGATGCCGAGCACGGAA-3' |
